# Development of a New Class of CXCR4-Targeting Radioligands Based on the Endogenous Antagonist EPI-X4 for Oncological Applications

**DOI:** 10.1021/acs.jmedchem.3c00131

**Published:** 2023-06-16

**Authors:** Raghuvir Haridas Gaonkar, Yannik Tim Schmidt, Rosalba Mansi, Yasser Almeida-Hernandez, Elsa Sanchez-Garcia, Mirja Harms, Jan Münch, Melpomeni Fani

**Affiliations:** † Division of Radiopharmaceutical Chemistry, Department Theragnostics, University Hospital Basel, Basel 4031, Switzerland; ‡ Computational Biochemistry, Center of Medical Biotechnology, University of Duisburg-Essen, Essen 45117, Germany; § Computational Bioengineering, Faculty of Bio- and Chemical Engineering, Technical University Dortmund, Dortmund 44227, Germany; ∥ Institute of Molecular Virology, Ulm University Medical Center, Ulm 89081, Germany; ⊥ Core Facility Functional Peptidomics, Ulm University Medical Center, Ulm 89081, Germany

## Abstract

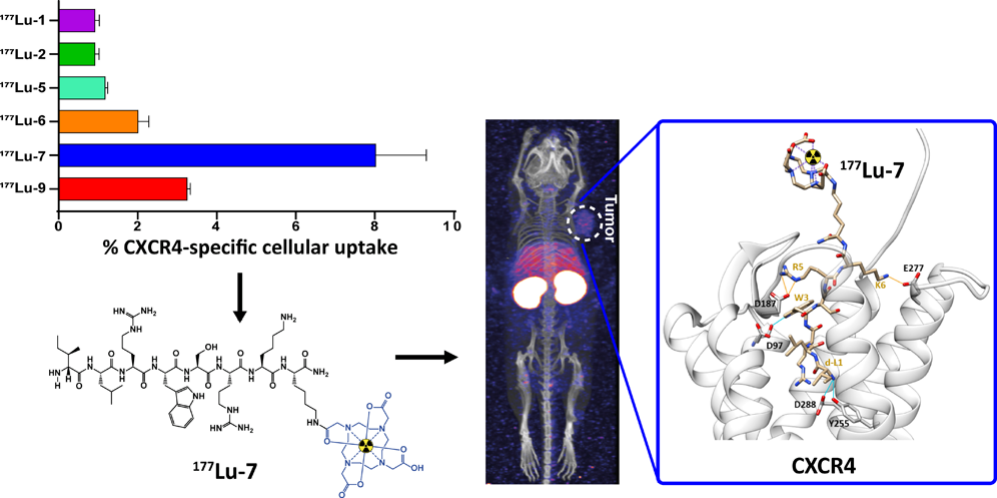

The peptide fragment of human serum albumin that was identified as an inhibitor of C–X–C motif chemokine receptor 4 (CXCR4), termed EPI-X4, was investigated as a scaffold for the development of CXCR4-targeting radio-theragnostics. Derivatives of its truncated version JM#21 (ILRWSRKLPCVS) were conjugated to 1,4,7,10-tetraazacyclododecane-1,4,7,10-tetraacetic acid (DOTA) and tested in Jurkat and Ghost-CXCR4 cells. Ligand-**1**, -**2**, -**5**, -**6**, -**7**, -**8**, and -**9** were selected for radiolabeling. Molecular modeling indicated that ^177^Lu-DOTA incorporation C-terminally did not interfere with the CXCR4 binding. Lipophilicity, *in vitro* plasma stability, and cellular uptake hinted ^177^Lu-**7** as superior. In Jurkat xenografts, all radioligands showed >90% washout from the body within an hour, with the exception of ^177^Lu-**7** and ^177^Lu-**9**. ^177^Lu-**7** demonstrated best CXCR4-tumor targeting. *Ex vivo* biodistribution and single-photon emission computed tomography (SPECT)/positron emission tomography (PET)/CT imaging of ^177^Lu-**7**/^68^Ga-**7** showed the same distribution profile for both radioligands, characterized by very low uptake in all nontargeted organs except the kidneys. The data support the feasibility of CXCR4-targeting with EPI-X4-based radioligands and designate ligand-**7** as a lead candidate for further optimization.

## Introduction

The C–X–C motif chemokine receptor 4 (CXCR4) belongs to the chemokine receptor family with pleiotropic functions under both physiological and pathological conditions.^1^ CXCR4 was initially discovered due to its ability to act as a coreceptor for HIV entry, while later it was found to be upregulated on a variety of solid and hematological neoplasms and associated with tumor growth and metastasis.^2,3^ In fact, of the 19.2 million newly reported cases of cancer in 2020, about 67% were CXCR4-related, which suggests CXCR4 as an important target for anticancer drug development.^4,5^ Today, several small molecules and peptidic CXCR4 antagonists and antibodies of CXCR4 are in preclinical and clinical development.^6^ Among them, Plerixafor (Mozobil, AMD3100) is the only approved CXCR4 antagonist so far. This compound is used to mobilize hematopoietic stem cells into the peripheral blood for autologous transplantation in cancer patients. Also, elevated CXCR4 expression has been associated with poor disease prognosis,^7,8^ which suggests imaging and quantification of CXCR4 levels to be clinically relevant for the management of patients with CXCR4-expressing malignancies.

Radio-theragnostics that specifically target CXCR4 are a valuable approach to cover both imaging and therapy of CXCR4-expressing malignancies. The field of radio-theragnostics has attracted enormous interest and investments in the last few years and has become an integral part of the modern armamentarium of precision and personalized medicine, for example, in prostate cancer and neuroendocrine tumor patients.^9,10^ The unique feature of radio-theragnostics is to use one substance for different medical applications: A ligand that specifically binds to a molecular target expressed on tumor cells or the tumor microenvironment can be (i) labeled with γ- or positron emitters for the detection of tumor lesions *via* single-photon emission computed tomography (SPECT) or positron emission tomography (PET), respectively, and (ii) labeled with β- or α-emitters for targeted radionuclide therapy. Among the radionuclides used, gallium-68 (a positron emitter for PET imaging) and lutetium-177 (a β-emitter for therapy, with a γ component) have clinically become the most successful theragnostic pair.

Development of CXCR4-targeting radio-theragnostics is an area of intensive research. The majority of such CXCR4-targeting radioligands developed so far are based on bicyclams, like AMD3100, on downsized peptides derived from T22, a synthetic derivative of the antimicrobial peptide polyphemusin II, like T140, or on cyclic pentapeptides based on FC-131.^11,12^ At present, the cyclic pentapeptide-based radioligands ^68^Ga-Pentixafor (cyclo(d-Tyr^1^-d-[NMe]Orn^2^(AMBS-(^68^Ga-DOTA))-Arg^3^-Nal^4^-Gly^5^) = ^68^Ga-DOTA-AMBS-CPCR4) and ^177^Lu-Pentixather (cyclo(3-iodo-d-Tyr^1^-d-[NMe]Orn^2^(AMBS-(^177^Lu-DOTA)-Arg^3^-Nal^4^-Gly^5^) = ^177^Lu-DOTA-AMBS-iodoCPCR4)) are the most advanced theragnostic pair for imaging and therapy, respectively, although still in the stage of clinical research.^13−15^ Unluckily, this theragnostic pair has certain limitations. The two radioligands showed notable differences in their biodistribution and pharmacokinetics.^16,17^ The iodination of Tyr in iodoCPCR4 significantly increased the lipophilicity of ^177^Lu-Pentixather, compared with ^68^Ga-Pentixafor. This resulted in enhanced plasma protein binding, delayed blood and body clearance, and enhanced hepatic accumulation. Furthermore, the entire CPCR4 core interacts with the binding pocket of CXCR4,^18^ leaving little room for the chemical modifications (*e.g.*, conjugation of chelators) and underlines the need of a more versatile scaffold. Very recently, a new cyclic peptide scaffold derived from the N-terminal region of the natural ligand CXCRL12 (Arg^29^–Glu^36^) by engineering^19,20^ showed encouraging results as a PET imaging agent when labeled with ^68^Ga.^21^ This new scaffold seems to be as well deeply embedded in the CXCR4 binding pocket, similar to CPCR4.^21^

In a study utilizing a peptide library derived from human hemofiltrate to search for new inhibitors of CXCR4-tropic HIV-1 infection, an endogenous antagonist of CXCR4, termed endogenous peptide inhibitor of CXCR4 (EPI-X4), was discovered (Table 1).^22,23^ EPI-X4 is a 16-mer fragment of human serum albumin that specifically binds to CXCR4. It blocks CXCL12-mediated signaling and suppresses chemokine-mediated effects, like cell migration and infiltration.^22,24^ In addition, EPI-X4 blocks basal receptor signaling and acts as an inverse agonist of CXCR4. Activity-optimized and truncated derivatives of EPI-X4 have recently been developed by rational drug design.^24^ Subsequent structure–activity relationship studies led to the 12-mer derivative EPI-X4 JM#21 (Table 1) with antagonistic activity in the nM range. This peptide has anti-inflammatory effects in mouse models of atopic dermatitis and eosinophilic asthma.^25^ However, a drawback of EPI-X4 JM#21 (and of the endogenous EPI-X4) is the low enzymatic stability in human plasma with a half-life of only 6 min (and 17 min, respectively).^25,26^ Since EPI-X4-derived peptides are primarily degraded in plasma on the N-terminal, corresponding modifications were introduced, which resulted in derivatives with significantly increased stability (*t*_1/2_ > 8 h). In addition, new lead derivatives were amidated at the C-terminus, which enabled further truncations.^24,27^

**Table 1:** Amino Acid Sequence of DOTA-Conjugated EPI-X4 Derivatives Based on the Lead Derivative JM#21 and Controls Used in the Study^a^

			IC_50_ value (nM ± SEM)^b^	
ligand	sequence	aa	ghost-CXCR4	jurkat
AMD3100			1186 ± 307	489 ± 88
EPI-X4	LVRYTKKVPQVSTPTL	16	4544 ± 1355	1779 ± 338
JM#21	I^1^LRWSRK^7^LPCVS^12^	12	183 ± 50	136 ± 49
**1**	I^1^LRWSRK^7^LPCVS^12^**K**(DOTA)	13	254 ± 58	303 ± 268
^nat^Lu-**1**			278 ± 103	208 ± 63
^nat^Lu-**1** dimer			225 ± 111	122 ± 63
**2**	I^1^LRWSRK^7^LPSVS^12^**K**(DOTA)	13	258 ± 108	172 ± 64
^nat^Lu-**2**			122 ± 45	49 ± 25
**3**	I^1^LRWSRK^7^LP**K**(DOTA)-NH_2_	10	98 ± 34	45 ± 15
**4**	I^1^LRWSRK^7^**K**(DOTA)-NH_2_	8	295 ± 86	161 ± ± 41
**5**	I^1^LRWSRK^7^(DOTA)-NH_2_	7	424 ± 120	120 ± 19
^nat^Lu-**5**			197 ± 62	113 ± 39
**6**	D-L^1^LRWSRK^7^LPCVS^12^**K**(DOTA)	13	747 ± 135	428 ± 127
^nat^Lu-**6**			1013 ± 233	465 ± 102
^nat^Lu-**6** dimer			763 ± 135	662 ± 160
**7**	D-I^1^LRWSRK^7^**K**(DOTA)-NH_2_	8	570 ± 152	433 ± 80
^nat^Lu-**7**			481 ± 106	435 ± 130
**8**	I^1^VRWSKK^7^(Pal)VPCS^12^**K**(DOTA)	12	15 ± 5	4 ± 2
^nat^Lu-**8**			30 ± 19	18 ± 5
**9**	D-L^1^LRWSRK^7^(E(Pal))**K**(DOTA)-NH_2_	9	6 ± 3	13 ± 5
^nat^Lu-**9**			6 ± 4	4 ± 1

a Bold indicates substitutions on the full sequence and underline indicates modifications or substitutions on the truncated version of JM#21. In all derivatives, DOTA is conjugated at the ε-NH_2_ of a lysine located at the C-terminus.

b IC_50_ values were obtained by competition with the CXCR4 antibody (clone 12G5); shown are data derived from at least three individual experiments; aa = amino acids, Pal = palmitic acid, AMD3100 = 1,1′-(1,4-phenylene bis(methylene))- bis-1,4,8,11-tetraazacyclotetradecane.

Taking into consideration that (a) the EPI-X4 derivatives are derived from an endogenous peptide that is highly specific for CXCR4^22^ and (b) newly developed derivatives are robust to degradation in blood plasma,^27^ we decided to investigate whether EPI-X4 could present a versatile scaffold for the development of novel CXCR4-targeting radio-theragnostics. To the best of our knowledge, this is the first time CXCR4 antagonists derived from this endogenous human peptide are used as a blueprint for the development of CXCR4-targeting radio-theragnostics. In the present study, based on EPI-X4 JM#21^25^ and stabilized derivatives,^27^ we selected a series of optimized derivatives and conjugated the chelator 1,4,7,10-tetraazacyclododecane-1,4,7,10-tetraacetic acid (DOTA). We complexed the DOTA conjugates (referred to as ligands) with natural lutetium (^nat^Lu) and labeled them with the radioisotope ^177^Lu. We then studied different *in vitro* and *in vivo* properties of these ^nat/177^Lu ligands using CXCR4-expressing cell lines and a CXCR4-expressing xenografted mouse model. The best performing ligand was labeled with ^68^Ga and compared *in vivo* with its ^177^Lu counterpart to verify the theragnostic concept.

## Results and Discussion

### Design of DOTA-Conjugated EPI-X4 Derivatives (Ligands)

The sequences of all of the EPI-X4 derivatives included in this study are represented in Table 1 and the analytical characterization of their DOTA conjugates is provided in Table S1 and Figures S1–S9. Since EPI-X4 and its derivatives bind *via* the N-terminus to the CXCR4 receptor,^24^ modifications for radiolabeling were introduced at the C-terminus. DOTA was conjugated *via* the ε-amino group of an additional lysine (K) residue coupled to the C-terminus (indicated in bold in Table 1), with the exception of the shortest truncated version of ligand-**5** in which the naturally occurring lysine was used. Other modifications or substitutions in the amino acid sequence of JM#21 are underlined in Table 1. Ligand-**1** bears the same sequence as the optimized JM#21,^25^ with the addition of K(DOTA) at the C-terminus. Ligand-**2** has a serine (S) instead of cysteine (C) at position 10, as cysteine might lead to oxidation and/or undesired conjugation in plasma, altering biodistribution and availability *in vivo*.^28^ Ligands **3**, **4**, and **5** are based on further truncated JM#21 derivatives in which the C-terminus is amidated in order to reduce electrostatic repulsion with the CXCR4 binding pocket.^24^ Ligands **6** and **7** have been modified at the N-terminus by the introduction of d-amino acids to increase resistance against enzymatic degradation.^27^ Ligand-**6** is based on ligand-**1** in which isoleucine (I) was substituted by d-leucine (d-L), and ligand-**7** is related to a truncated version of ligand-4 in which I was substituted by d-isoleucine (d-I). Furthermore, two additional derivatives were included, ligand-**8** and ligand-**9**, bearing palmitic acid in position 7 (among other modifications) either directly *via* the side chain of lysine (ligand-**8**) or *via* a glutamic acid linker (ligand-**9**). The rational is based on indications that peptide palmitoylation increases the circulation time *in vivo* due to the interaction with plasma serum albumin.^29,30^ In the context of imaging, this property may not be favorable. However, in the context of radionuclide therapy, radioligand binding to albumin *via* albumin-binding moieties, including palmitic acid, has shown certain advantages.^31^ Therefore, in the present study, we asked the question, if palmitoylation could be beneficial for this class of radioligands, as it might prolong circulation of the variants in the blood and, thus, improve tumor targeting and residence time, a strategy used with other radiotherapeutics.^31−33^

### Functional Characterization

Binding of the DOTA-conjugated EPI-X4 derivatives to CXCR4 was assessed by an antibody-competition assay^26^ using Ghost cells stably transfected with CXCR4, as well as on Jurkat cells, which are naturally expressing high levels of CXCR4. This assay is based on the competition of orthosteric CXCR4 ligands with the anti-CXCR4-antibody clone 12G5, which binds to the second extracellular loop of the receptor. The results are shown in Table 1 and detailed in Figures S10 and S11. The wild-type EPI-X4 was used as an internal control and AMD3100 was applied as a reference ligand.

The optimized EPI-X4 derivative JM#21 competed with the antibody with half-maximal inhibitory concentration (IC_50_) values of 183 nM in Ghost-CXCR4 cells and 136 nM in Jurkat cells, respectively, and was more active than the wild-type peptide EPI-X4, confirming previous results^25^ (Table 1). For the DOTA-conjugated EPI-X4 derivatives, a wide range of IC_50_ values was observed (6–747 nM in Ghost-CXCR4 cells and 4–433 nM in Jurkat cells) with a strong correlation among them (Supporting Figure 12). In comparison to the lead compound JM#21, DOTA conjugation slightly decreased the activity (ligand-**1** and -**2**). C-terminal truncation, in combination with DOTA conjugation and amidation, was tolerated in terms of CXCR4 affinity (ligand-**4** and ligand-**5**), and in some cases even an increased activity (ligand-**3**), compared to JM#21, could be observed. N-terminal substitution with d-amino acids (ligand-**6** and ligand-**7**) led to on average 2-fold decreased activity compared to their l-version counterparts. For ligand-**8** and ligand-**9**, the conjugation of palmitic acid led to an increased affinity with IC_50_ values reaching a single-digit nanomolar range.

Among the nine tested DOTA conjugates, seven were selected for exploring the feasibility of CXCR4 targeting with EPI-X4-based radioligands. These were ligand-**1** and ligand-**2** based on their similarity to the lead JM#21. Ligand-**5**, the shortest identified active derivative as a representative of the subgroup with the three derivatives with amidated C-terminus (ligand-**3**, ligand-**4**, and ligand-**5**), ligand-**6**, and ligand-**7** due to their higher stability in human plasma,^27^ and ligand-**8** and ligand-**9** due to their high activity and potential benefit regarding prolonged blood circulation.

### Complexation with Lutetium (^nat^Lu) and Lutetium-177 (^177^Lu)

^nat^Lu complexes were synthesized with a yield of 70–80% and a purity of >95% (Table S2 and Figures S13–S23) except for the cysteine-containing ligands (-**1** and -**6**), which exhibited two species (2 peaks) in the UV chromatogram (Figures S13 and S18). To identify these peaks, matrix-assisted laser desorption ionization time-of-flight (MALDI-TOF) MS analysis was performed, which along with a monomer (Figures S14 and S19) indicated dimerization (Figures S15 and S20) of the Cys-containing peptides by the formation of intermolecular disulfide bonds. Consequently, ^nat^Lu-**1**, ^nat^Lu-**6**, and the respective dimers were isolated by semipreparative reverse-phase high-performance liquid chromatography (RP-HPLC). Complexation of ligand-**8** was performed in the presence of dithiothreitol (DTT), and in this case, no dimerization was observed.

Ligand dimerization was also observed during the ^177^Lu labelings; however, in this case, separation was not feasible. Thus, all stock solutions of Cys-containing peptides were prepared in the presence of DTT (10 mM), and the temperature during radiolabeling was reduced from 95 to 75 °C to prevent the formation of disulfide bonds, resulting in a single radioactive species.

Overall, reproducible ^177^Lu labelings were obtained for all tested ligands with radiochemical purity (RCP) > 95%, except for ligand-**8**. ^177^Lu-**8** displayed radiochemical yield (RCY) and RCP < 50% (Supporting Figure 24). Furthermore, ^177^Lu-**8** was sticking to the HPLC column and injector. Therefore, the results of the radio-HPLC analysis were inconclusive. The quality control was shifted from radio-HPLC to thin layer chromatography (TLC) and read-out in a γ-counter. The quality control displayed good RCY, but RCP could not be traced for this radioligand and hence ^177^Lu-**8** was excluded from further evaluation. ^177^Lu-**9** showed a similar problem during radio-HPLC analysis, but optimization of the quality control methods (gradient) of ^177^Lu-**9** led to reliable and acceptable RCP and RCY, allowing further evaluation.

The radioligands were prepared at apparent molar activities of 5–80 MBq/nmol, depending on the type of experiment performed.

### CXCR4 Binding

Metalation can affect ligand architecture and, thus, receptor affinities. Therefore, we tested and compared all ligands after complexation with ^nat^Lu for receptor competition with the CXCR4-antibody clone 12G5 (Table 1 and detailed in Figures S10 and S11). For most ligands, ^nat^Lu incorporation had no or only minor impact on receptor binding (ligand-**1**, ligand-**6**, ligand-**7**, and ligand-**9**), while in certain cases, *i.e.*, ^nat^Lu-**2** and ^nat^Lu-**5**, metalation even led to an activity increase compared to the free ligands. Once again, the two palmitic acid derivatives (^nat^Lu-**8** and ^nat^Lu-**9**) showed the lowest IC_50_ values, indicating a contribution of the hydrophobic lipid on the CXCR4 recognition and affinity, with ^nat^Lu-**9** being the most potent one. Dimerization did not notably impact the activity of the ^nat^Lu ligands and therefore the dimers were not considered for further evaluation.

**Figure 1:**
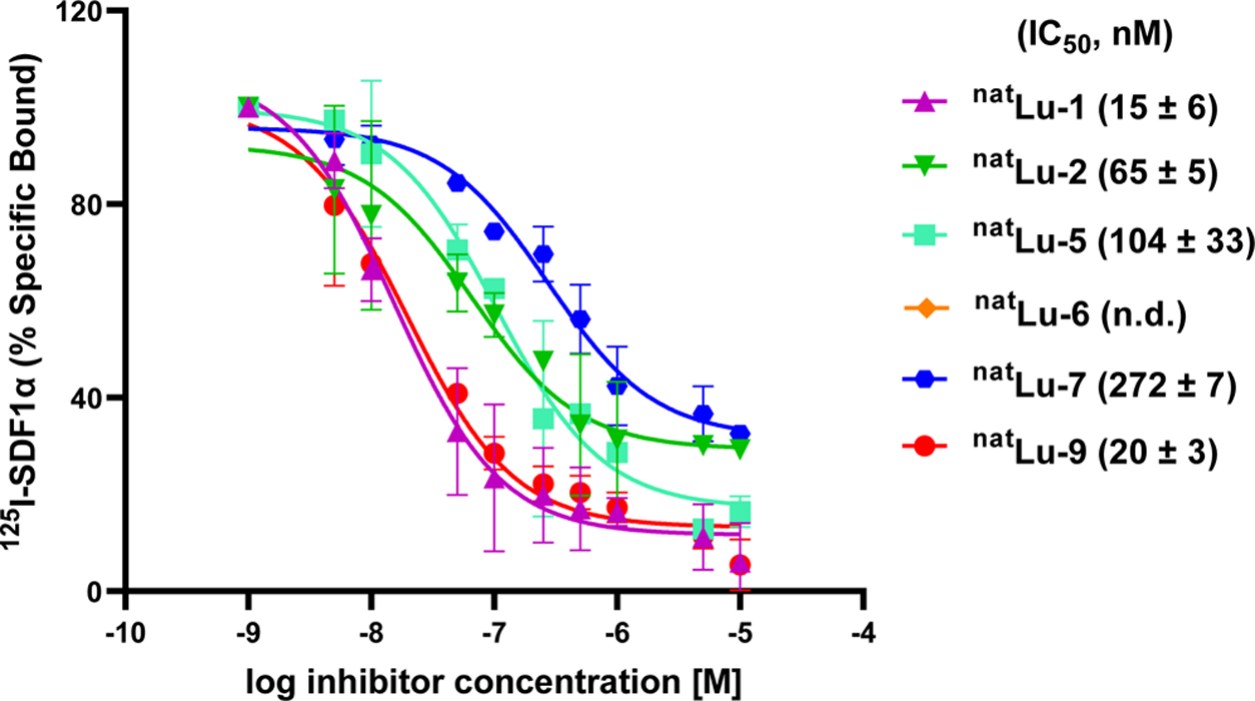
Competition binding curves displaying the effect of increasing concentrations of the ^nat^Lu ligands on the binding of [^125^I]SDF-1α (displacement) in Jurkat cells.The data are derived from at least two individual experiments, each in triplicate and are expressed as mean ± SD. n.d.: Not determined.

The IC_50_ values of the ^nat^Lu ligands were also determined by competing with [^125^I]SDF-1α (stromal-derived factor 1α = CXCL12) in Jurkat cells (Figure 1). Overall, the affinity of the ^nat^Lu ligands showed a very similar trend to that of the antibody-competition assay, with the exception of ^nat^Lu-**1**. ^nat^Lu-**1** displayed very high affinity, similar to ^nat^Lu-**9**, in the competition with the natural ligand, contrary to what was seen in the competition with the antibody. The affinity trend for all other ^nat^Lu ligands was the same in both assays, i.e., ^nat^Lu-**9** > ^nat^Lu-**2** > ^nat^Lu-**5** > ^nat^Lu-**7**. Different IC_50_ values between the two assays for the same ligand were due to different competitors (12G5 antibody and SDF-1α, respectively), while the reason for the discrepancy regarding ^nat^Lu-**1** still remains unclear.

### Shelf-Life and Log *D*

The shelf-life of each ^177^Lu-labeled ligand was monitored at room temperature up to 24 h after radiolabeling. The RCP and stability data are summarized in Table 2.

**Table 2:** Radiochemical Purity of ^177^Lu Ligands at Room Temperature at Different Time Points after Radiolabeling^a^

radiochemical purity (%)						
time (h)	^177^Lu-**1**	^177^Lu-**2**	^177^Lu-**5**	^177^Lu-**6**	^177^Lu-**7**	^177^Lu-**9**
0	98 ± 0	97 ± 0	99 ± 0	98 ± 1	98 ± 1	96 ± 3
1	93 ± 3	97 ± 0	98 ± 0	96 ± 3	95 ± 0	96 ± 2
2	92 ± 6	96 ± 1	97 ± 1	92 ± 9	94 ± 0	94 ± 1
4	90 ± 7	96 ± 1	95 ± 1	90 ± 9	88 ± 3	93 ± 1
24	62 ± 13	78 ± 1	80 ± 2	74 ± 1	66 ± 0	80 ± 10

a Analysis was performed by radio-HPLC of *n* = 3 independent radiolabelings per ligand.

All of the radioligands were >90% stable at 2 h, which remained unchanged even after 4 h. The most stable radioligands were ^177^Lu-**2**, ^177^Lu-**5**, and ^177^Lu-**9** with approx. 80% remaining intact after 24 h at room temperature, whereas ^177^Lu-**1** and ^177^Lu-**7** remained approx. 60% intact at this time point. The radiochemical species observed after 24 h, in addition to the intact radioligand, were attributed to the formation of radiolytic byproducts. These byproducts depend on the chemical structure (peptide sequence) of the ligand, which explains the difference among them. Radiolysis can be possibly diminished with the use of radiolytic scavengers after or during radiolabeling. No release of ^177^Lu from the ^177^Lu-DOTA complex was detected for any of the radioligands over time.

**Figure 2:**
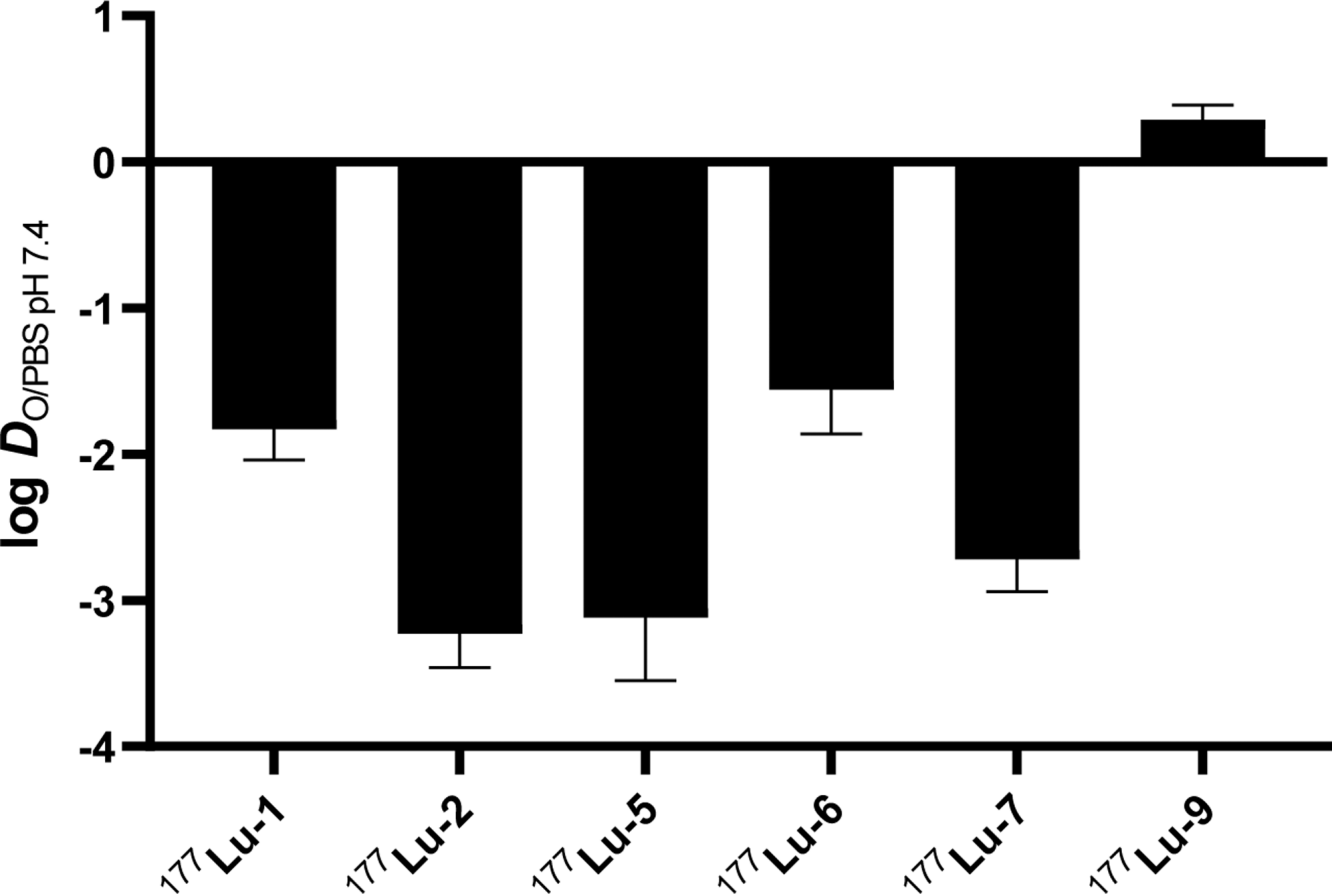
Lipophilicity of the ^177^Lu-labeled ligands was assessed by the determination of their distribution (log *D*) between octanol (O) and phosphate-buffered saline (PBS) at the physiological pH of blood serum, pH = 7.4.

The radioligands displayed a broad spectrum of polar character (Figure 2). ^177^Lu-**1** and ^177^Lu-**2** differing only in one amino acid at position 10 (cysteine and serine, respectively) showed substantially different lipophilicities. The serine derivative ^177^Lu-**2** was the most hydrophilic among all tested radioligands (log *D*_O/PBS pH7.4_ = −3.23 ± 0.23), similar to the shortest variant ^177^Lu-**5**. The introduction of d- for l-amino acids in the derivatives ^177^Lu-**6** and ^177^Lu-**7** did not contribute to their lipophilicity (see ^177^Lu-**6***vs*^177^Lu-**1**). As expected, ^177^Lu-**9** bearing a hydrophobic fatty acid side chain showed the highest hydrophobicity, exemplified by the positive log *D* value (log *D*_O/PBS pH7.4_ = 0.29 ± 0.10).

### *In Vitro* Metabolic Stability and Plasma Protein Binding

**Figure 3:**
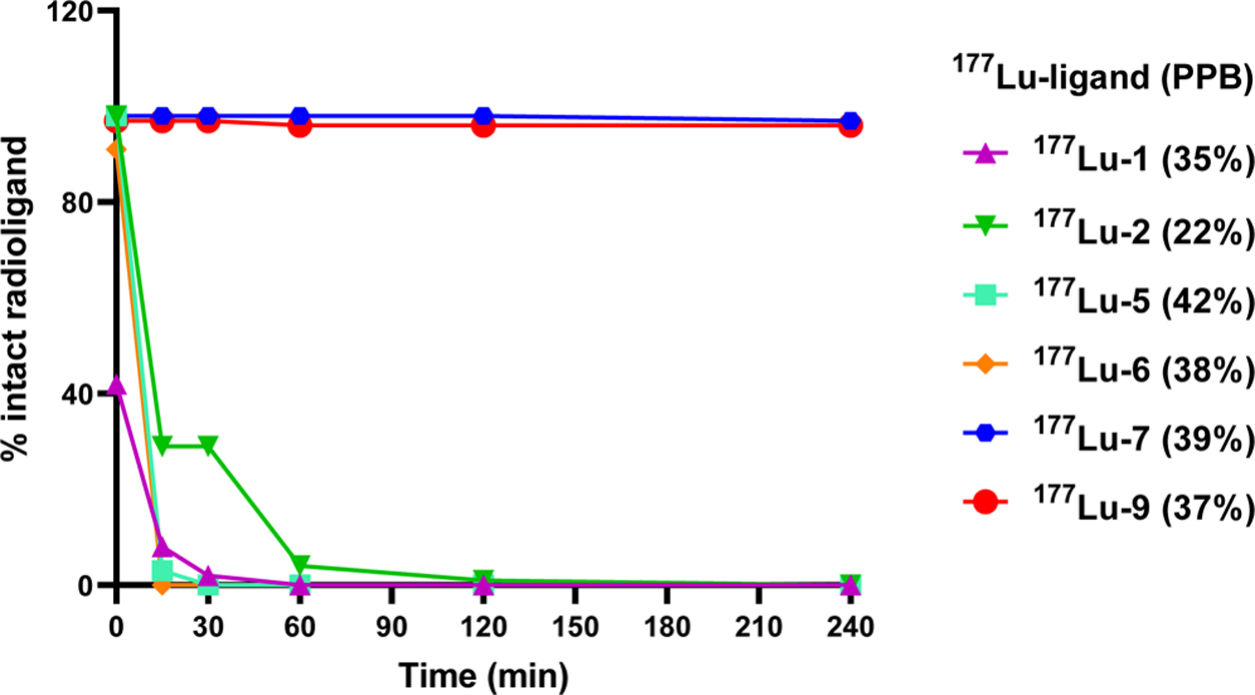
Stability of the ^177^Lu ligands in human plasma at 37 °C, expressed as % of intact radioligand over time, assessed by radio-HPLC. The % of plasma protein binding (PPB) after 60 min incubation is provided in parenthesis and it is expressed as the % of radioligand in the protein fraction (pellet) versus the total activity in the plasma.

The stability and protein binding of all of the ^177^Lu ligands were assessed in fresh human plasma (Figure 3). ^177^Lu-**1**, ^177^Lu-**5**, and ^177^Lu-**6** were completely degraded within 15 min of incubation, while ^177^Lu-**2** remained ∼30% intact in plasma up to 30 min, followed by complete degradation after 60 min. ^177^Lu-**7** and ^177^Lu-**9** were the only radioligands that displayed high metabolic stability in plasma up to 4 h. Representative radiochromatograms are provided in Figure S25 for ^177^Lu-**1**, ^177^Lu-**2**, and ^177^Lu-**7**. Plasma protein binding (PPB) was similar and moderate for all ^177^Lu ligands, as reported in Figure 3. This included ^177^Lu-**9** despite its fatty acid chain. All radioligands showed a significantly lower level of PPB compared to ^177^Lu-Pentixather (97%).^17^

### Cellular Uptake

**Figure 4:**
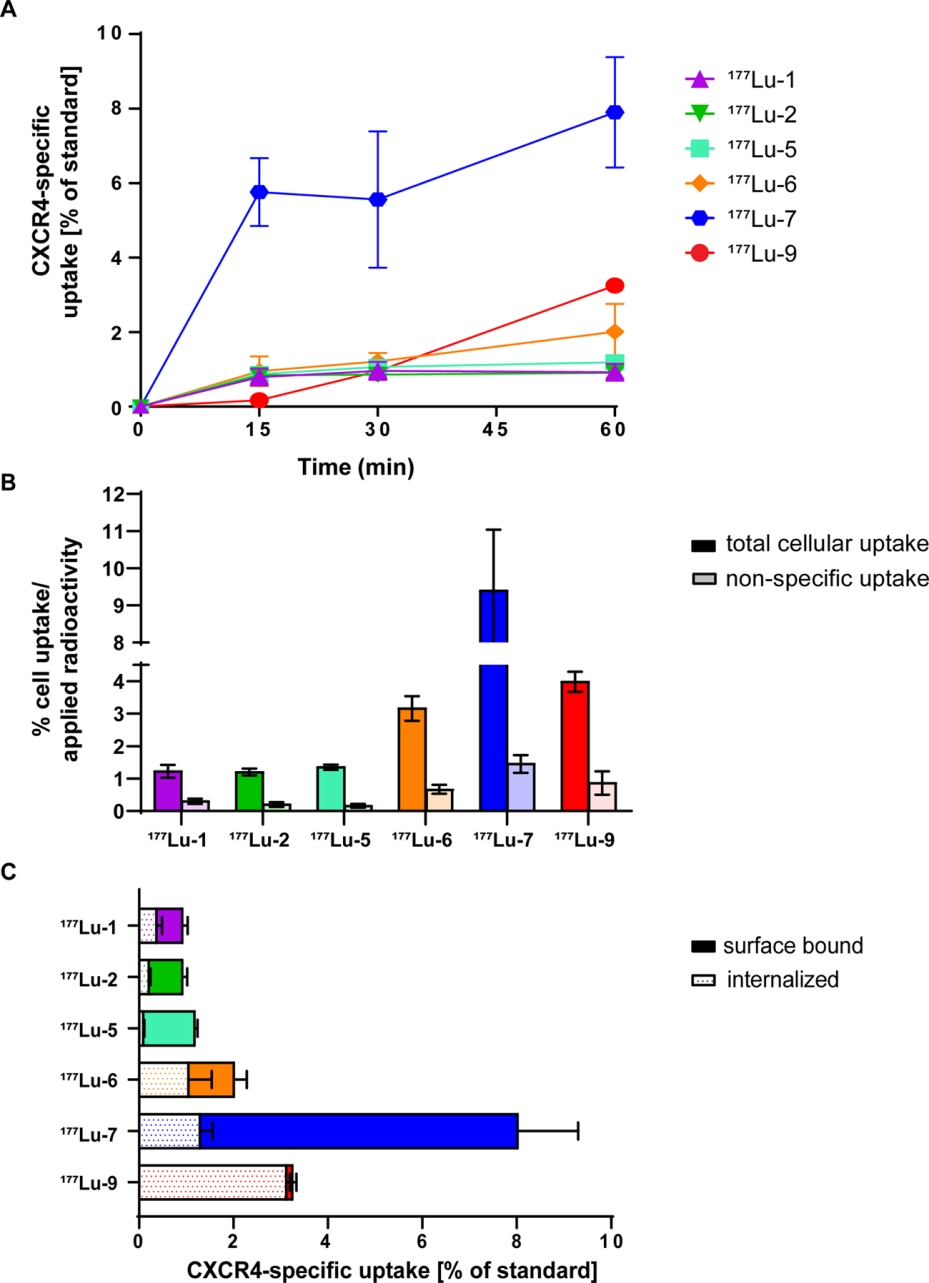
*In vitro* assessment in Ghost-CXCR4 cells at 37 °C. (A) Cellular uptake (surface-bound and internalized) of ^177^Lu ligands over time, (B) cellular uptake expressed as % of the applied activity of ^177^Lu ligands in the absence (dark bars—total uptake) and presence (light bars—nonspecific uptake) of AMD3100 after 60 min, and (C) distribution of ^177^Lu ligands in membrane-bound and internalized fractions in Ghost-CXCR4 cells after 60 min. Each experiment was performed in triplicate. Results are means ± SD [% of applied activity] from a minimum of two separate experiments.

Cellular uptake and distribution of the ^177^Lu-labeled ligands were assessed in Ghost-CXCR4 cells at 37 °C from 15 min up to 1 h. The uptake was found to be CXCR4-mediated and time-dependent (Figure 4). While the majority of the ^177^Lu ligands exhibited a cellular uptake of around 1% of the applied activity, ^177^Lu-**7** and ^177^Lu-**9** displayed a higher cellular uptake at 60 min (7.90 ± 1.48 and 3.25 ± 0.06%, respectively, Figure 4A). Interestingly, the results of the cellular uptake assays were not in agreement with the affinity data. More specifically, ^177^Lu-**1** and ^177^Lu-**2** showed higher affinity in the antibody-competition assay than ^177^Lu-**5**, ^177^Lu**-6**, and ^177^Lu**-7**, but they performed worse in the cellular uptake assay in Ghost-CXCR4 cells (Figure 4A). In addition, the high uptake of ^177^Lu-**7**, among all radioligands, could not be predicted from the affinity data.

This difference could be partly explained by the very different experimental settings. In the affinity assay, the ligands were competing with the 12G5 antibody for binding to CXCR4. Apparently, this competition assay is not predictable for internalization. On the other hand, the cellular uptake assay is based on the direct interaction of the radioligand with the receptor expressed on the cell membrane. This uptake was proven to be CXCR4-mediated by blocking studies, where the nonspecific binding (Figure 4B) was determined in the presence of a very high excess of AMD3100.^34^ We considered that the cellular uptake values are more relevant for the purpose of our study.

In terms of subcellular distribution, all tested radioligands were mainly bound on the cell surface, with only small part of it getting internalized (Figure 4C). Thus, all radioligands seem to act as antagonists of CXCR4. The only exception was ^177^Lu-**9** that is almost entirely internalized. We hypothesize that the fatty acid chain is mainly responsible for this behavior as it might increase affinity toward the cell membrane and enhance intracellular distribution.^35,36^

Among all radioligands tested, ^177^Lu-**7** had an advantage by means of distinguished highest CXCR4-mediated cellular uptake *in vitro*.

### Molecular Modeling of Lu-7

To gain more insights into the effect of the ^177^Lu-DOTA group on the binding of the EPI-X4 derivative of ligand-**7**, we built models of Lu-**7** in complex with CXCR4, based on a previous structure of the JM#173/CXCR4 complex.^27^ JM#173 has the same peptide sequence as ligand-**7** without the chelator DOTA. The structure of the JM#173/CXCR4 complex was obtained by molecular docking and Gaussian accelerated molecular dynamics simulations (GaMD). Using the JM#173/CXCR4 model as a basis, we first attached, *via* an amide bond, a C-terminal amidated Lys residue with Lu^3+^-DOTA to the Nε atom of Lys^6^ of JM#173. The DOTA coordinates, in which we replaced Gd by Lu, were extracted from the structure reported in the Protein Data Bank with ID 1NC4.^37^ The resulting model was subjected to a docking and optimization protocol (see Computational Details).

**Figure 5:**
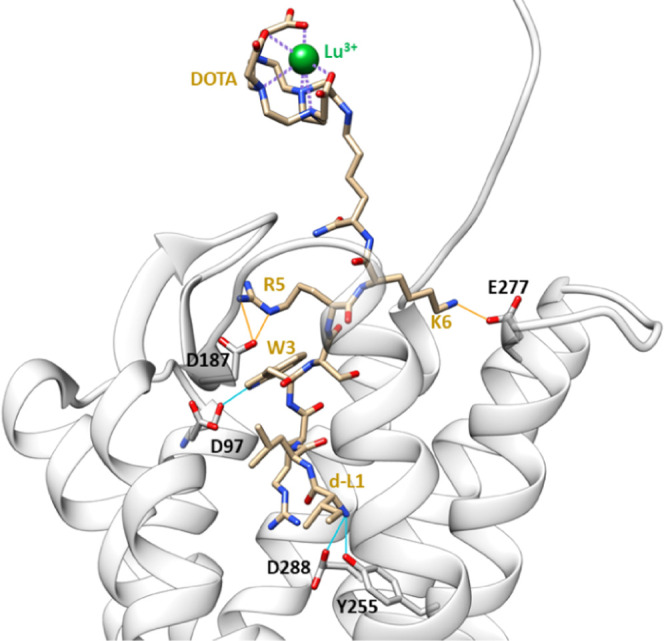
Model of CXCR4 in complex with Lu^3+^-**7**. Blue bonds show H-bonds and orange bonds indicate salt bridges. For the CXCR4 model, see the Molecular Modeling Section in Material and Methods Section.

Our optimized model shows that the C-terminal Lys acts as a spacer, exposing the DOTA group to the solvent and avoiding DOTA’s steric hindrance with CXCR4. In the Lu-**7**/CXCR4 complex, the peptide–protein interactions are not affected by the presence of the Lys-DOTA-Lu^3+^ group (Figure 5) and remain similar to those established between JM#173 and CXCR4. As shown in Figure 3, Lu-7 interacts *via* hydrogen bonding with D97, Y255, and D288 of CXCR4, involving the residues W3 and d-L1 of the peptide. Furthermore, R5 and K6 of the peptide establish salt bridges with the acidic residues D187 and E277 of CXCR4, respectively.

### *In Vivo* Evaluation of Radioligands in Jurkat Xenografts

#### SPECT/CT and PET/CT Imaging

**Figure 6:**
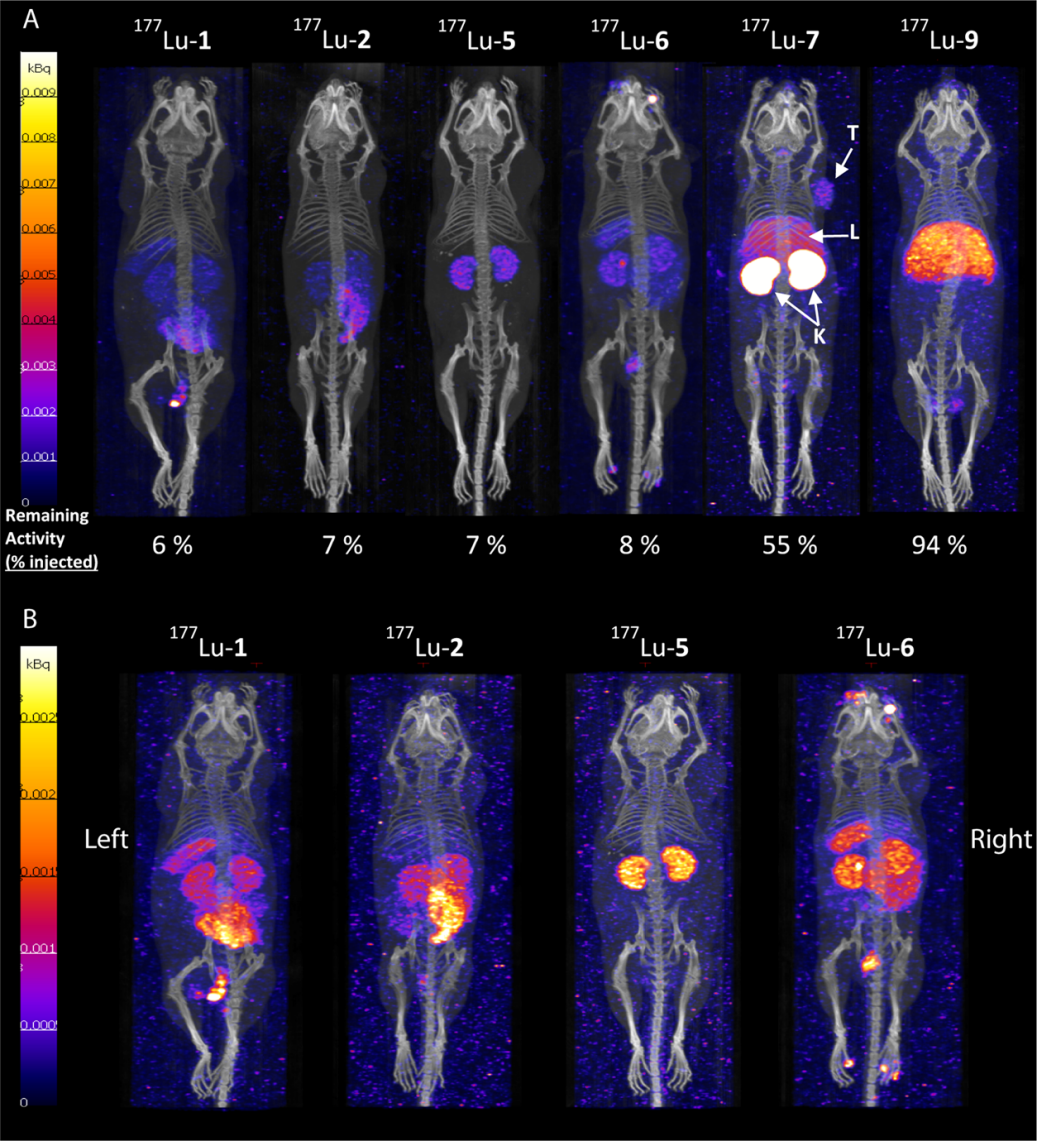
SPECT/CT images as maximum intensity projections (MIPs) of Jurkat tumor-bearing mice at 1 h p.i. of ^177^Lu-labeled ligands (200 pmol, ∼15 MBq). (A) Images have been adjusted in the same scale for direct comparison, even though the remaining activity in the body of the mice after 1 h (% of the remaining activity is mentioned in the images) differs significantly among the radioligands due to the differences in the total body clearance. (B) Same SPECT/CT images of ^177^Lu-**1**, ^177^Lu-**2**, ^177^Lu-**5**, and ^177^Lu-**6**, adapted in a lower scale. T: tumor, L: liver, and K: kidneys. Images are displayed from posterior (left and right are indicated).

The ability of the ^177^Lu ligands to target CXCR4 *in vivo* was assessed by SPECT/CT imaging in Jurkat xenografts. Figure 6 shows representative SPECT/CT images following the administration of each ^177^Lu ligand (100 μL, 200 pmol, 12–16 MBq). 1 h after injection, the remaining amount of radioligand in the body (and *vice versa*, the washout) was quantified by measuring the total body activity (mouse) in a dose calibrator. The % of the radioligand remaining in the body after 1 h is reported in Figure 6A as % of the remaining activity in reference to the injected activity (100%). Figure 6A shows SPECT/CT images of all radioligands, in comparison, using the same scale, while Figure 6B shows the same SPECT/CT images of ^177^Lu-**1**, ^177^Lu**-2**, ^177^Lu**-5**, and ^177^Lu**-6**, adapted in a lower scale.

The first two radioligands ^177^Lu-**1** and ^177^Lu-**2** were rapidly washed out, with only 6–7% of the radioligand retained in the body at 1 h p.i. Such fast washout suggests high blood and body clearance and/or metabolic instability, followed by fast excretion of the metabolites. In the cases of ^177^Lu-**1** and ^177^Lu-**2**, we presume that the fast washout is mainly due to their rapid metabolic degradation as they are both structurally very similar to JM#21, which has a half-life of only ∼6 min in plasma.^25^ This is supported by the *in vitro* metabolic stability data, demonstating high instability (Figure 3). The two radioligands showed very similar, but not identical, biodistribution.^177^Lu-**1** accumulated in the spleen, which was not the case for the serine derivative ^177^Lu-**2** (Figure 6B). This might be attributed to the more lipophilic character of ^177^Lu-**1**, compared to ^177^Lu-**2**, and/or to the ability to covalently bind to plasma proteins. In fact, the plasma protein binding was found higher for ^177^Lu-**1** than for ^177^Lu-**2** (35 *vs* 22%, respectively, after 60 min incubation in human plasma). However, lipophilicity seems to be more decisive. This is evident by the differences seen between ^117^Lu-**5** and ^177^Lu-**6**, having the same PPB (∼40%), with the more lipophilic ^177^Lu-**6** also accumulating in the spleen but not the hydrophilic ^177^Lu-**5**. This shortest version carrying C-terminal amidation (often used for stabilization purposes) however showed the same rapid washout from the body. Only 7% remained after 1 h, most probably due to fast degradation^24^ and fast clearance, as the result of the small size and hydrophilicity. ^177^Lu-**5** was the only ligand that did not accumulate in any other organ but the kidneys, supporting renal clearance. The same biodistribution profile was also observed for ^177^Lu-**3** (Figure S26), which belongs to the “subgroup” of the C-terminus amidated truncated derivatives (*i.e.*, ligand-**3**, ligand-**4**, and ligand-**5**). As discussed above, the introduction of a d-amino acid at the vulnerable N-terminus of the EPI-X4 derivatives increases stability *ex vivo* in human plasma^27^ and it was expected to improve the stability *in vivo*. However, for the d-l variant ^177^Lu-**6**, only 8% of the activity remained after 1 h, similar to ^177^Lu-**1** with l-I at position 1, which is in line with the low stability found in plasma. The two radioligands demonstrated a very similar biodistribution profile, with the d-amino acid modestly impacting the spleen and kidney uptake (increase) and the intestinal uptake (decrease).

**Figure 7:**
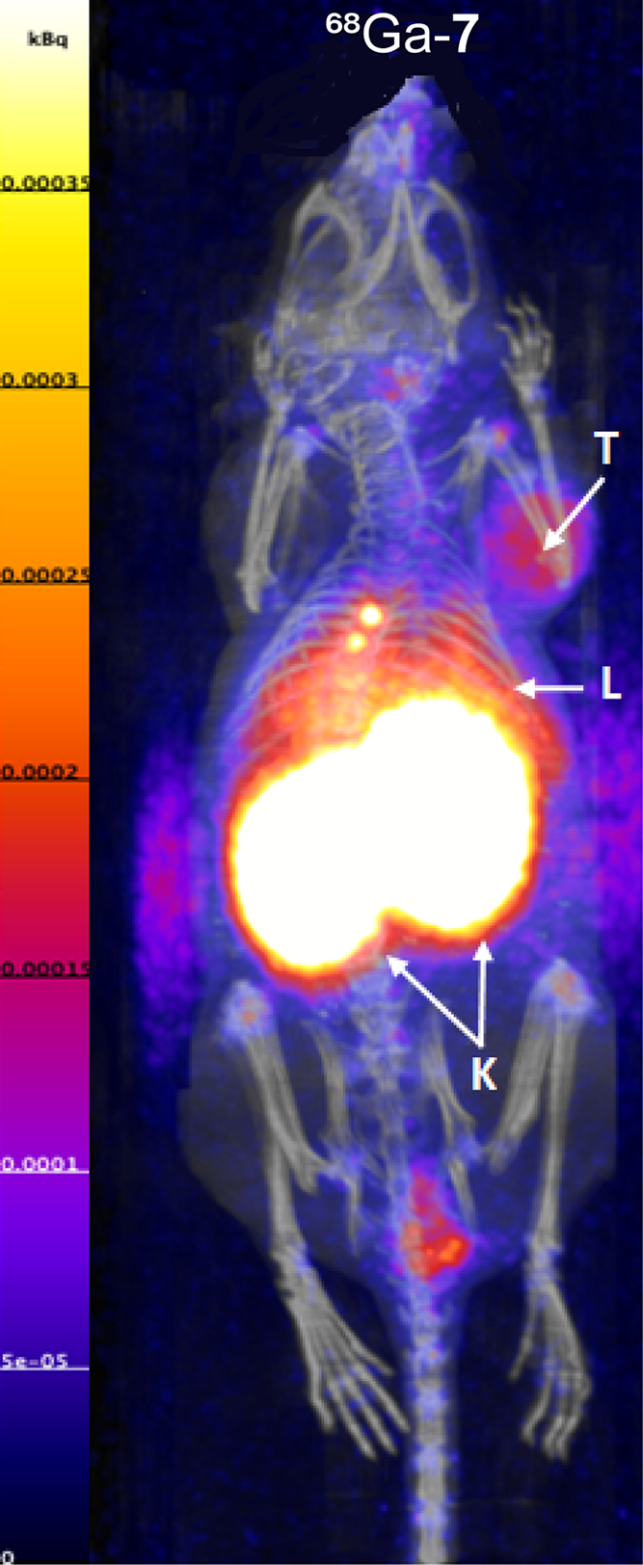
PET/CT images as maximum intensity projections (MIPs) of Jurkat tumor-bearing mice at 1 h p.i. of ^68^Ga-**7** (200 pmol, 8 MBq).

A substantial difference in the washout was found for ^177^Lu-**7**, which remained in the body approx. 55% after 1 h. The total body distribution of ^177^Lu-**7** is characterized by the high accumulation in the kidneys, indicating the urinary system as the main excretory pathway. Possibly, this is the case due to its hydrophilic character. Its low hepatic uptake might be attributed, at least partially, to CXCR4 expression in the liver.^38^^177^Lu-**7** accumulated and visualized the CXCR4-expressing tumor xenograft *via* SPECT/CT imaging. Similarly, ^68^Ga-**7** visualized the CXCR4-expressing tumor xenograft *via* PET/CT imaging (Figure 7) and showed the same biodistribution as ^177^Lu-**7**. The total body distribution of ^177^Lu-**7**/^68^Ga-**7** suggests improved metabolic stability *in vivo*, compared to all other ligands. This is supported by the results of the *in vitro* plasma stability studies. This enhanced stability is thought to be the result of the protection to enzymatic degradation on both the N-terminus with d-I and C-terminus *via* amidation.^27^

Contrary to all other radioligands, ^177^Lu-**9** showed almost no excretion from the body, with 94% remaining after 1 h. However, ^177^Lu-**9** accumulated almost entirely in the liver, which can be explained by its lipophilic character due to the fatty acid chain, while it failed to visualize CXCR4-expressing tumors *in vivo*.

Peptide lipidation is often used as a strategy to improve the pharmacokinetics and potency of peptide therapeutics.^35^ Recently, this strategy was used for improving the therapeutic efficacy of radioligands targeting the tumor microenvironment *via* fibroblast activation protein (FAP).^31^ It was shown that despite the higher background activity and liver uptake of the radioligand bearing palmitic acid, the accumulation in the tumor was high and persistent, compared to the nonlipidated counterpart. However, here we show that this is not an ideal strategy for EPI-X4-based radiotherapeutics. Given that the application is mainly—if not exclusively—systemic, such a lipophilic molecule ends up quickly in the liver. Limited systemic circulation reduces the chance to bind to the target of interest. In many cancers, the liver is a common site of metastasis, and a radio-theragnostic that accumulates unspecifically usually fails in the image contrast and will unnecessarily irradiate the entire liver. Therefore, ^177^Lu-**9** was excluded from further analyses and lipidation using large fatty acids rejected as a strategy for further optimization.

Finally, assessing the *in vivo* profile of all radioligands, ^177^Lu-**7** was selected for *in vivo* quantification, in parallel with its imaging counterpart, ^68^Ga-**7**.

### Quantitative Biodistribution of ^177^Lu-7/^68^Ga-7

Quantification of the total body distribution of ^177^Lu-**7** and ^68^Ga-**7** was performed in Jurkat xenografted mice after intravenous injection of 100 μL, 200 pmol, with 1 and 5 MBq, respectively (*n* = 5). The results are reported in Table 3. The mice were sacrificed at 1 h p.i. ^177^Lu-**7** and ^68^Ga-**7** accumulated mainly in the kidneys (83.1 ± 25.5 and 75.6 ± 30.3%IA/g, respectively, *p* = 0.59), liver (5.19 ± 1.08 and 4.08 ± 1.05%IA/g, respectively, *p* = 0.07) and tumor (2.25 ± 0.43 and 2.24 ± 0.57%IA/g, respectively, *p* = 0.97), as already seen in the SPECT/CT and PET/CT images (Figures 6 and 7). Uptake in the tumor was lower than for the kidneys and liver but higher as in all other tissues investigated. However, the accumulation in the tumor depends greatly on the level of the CXCR4 expression and can vary significantly among different animal models. The uptake seen in the liver might be attributed, at least partially, to the fact that CXCR4 is constitutively expressed in the sinusoidal endothelial cells of the murine liver^38^ or could be related to another yet-unknown mechanism previously reported for other CXCR4-targeting probes.^11,39^ Regretfully, there are no direct measurements available for the affinity of ^177^Lu-**7** with the murine CXCR4 (mCXCR4). This is relevant for assessing its total body distribution. However, we have shown that EPI-X4 and EPI-X4 derivatives are active in mouse models of inflammatory diseases^22,25^ and cancer.^40^ More specifically, EPI-X4 derivatives such as JM#21 reduced immune cell influx in lungs of mice suffering from allergic asthma or reduced skin inflammation in a murine model of atopic dermatitis upon topical administration. These results suggest that EPI-X4 and its derivatives in general can antagonistically bind to mCXCR4, thereby exhibiting anti-inflammatory characteristics. Furthermore, we have shown by molecular dynamic simulations that JM#173 exhibits the same binding mechanism as JM#21.^27^ Thus, we speculate that the DOTA conjugate of JM#173 (ligand-**7**) also cross-reacts to the mCXCR4. Nevertheless, we still do not know to which extent ligand-**7** binds to mCXCR4. Last, but not the least, the uptake in the kidneys was predominant, and given the highly hydrophilic character of ^177^Lu-**7**, is attributed, at least partially, to its excretion *via* this path.

**Table 3:** Biodistribution Data of ^177^Lu/^68^Ga-**7** in Jurkat Xenografted Nude Mice at 1 h p.i. (*n* = 5)^a^

organ	^177^Lu-7	^177^Lu-7 + AMD3100	^68^Ga-7
blood	0.42 ± 0.12	0.38 ± 0.13	0.52 ± 0.14
heart	0.21 ± 0.05	0.22 ± 0.08	0.25 ± 0.09
lung	0.68 ± 0.11	0.59 ± 0.13	1.78 ± 0.81
liver	5.19 ± 1.08^b^	0.90 ± 0.23^b^	4.08 ± 1.05
pancreas	0.14 ± 0.02	0.13 ± 0.03	0.20 ± 0.11
spleen	0.81 ± 0.16^c^	0.27 ± 0.05^c^	0.85 ± 0.29
stomach	0.30 ± 0.06	0.41 ± 0.26	0.41 ± 0.16
intestine	0.37 ± 0.10	0.25 ± 0.07	0.59 ± 0.18
adrenal	1.09 ± 0.42	0.94 ± 0.78	2.69 ± 0.94
kidney	83.1 ± 25.5^d^	22.2 ± 7.29^d^	75.6 ± 30.3
muscle	0.27 ± 0.14	0.12 ± 0.02	0.44 ± 0.26
femur	1.06 ± 0.28	0.68 ± 0.36	1.29 ± 0.43
tumor	2.25 ± 0.43^e^	0.87 ± 0.43^e^	2.24 ± 0.57

a The results are expressed as the % injected activity per gram of organ (% IA/g). Statistical analysis was performed by Welch’s *t*-test and *p* values < 0.05 are considered statistically significant.

b *p* < 0.0001.

c *p* < 0.0001.

d *p* < 0.0001.

e *p* = 0.0018.

The biodistribution profile of ^177^Lu-**7**, which is based on a new CXCR4-targeting scaffold, displayed certain favorable features compared to the most advanced CXCR4-targeting radioligand ^177^Lu-Pentixather. ^177^Lu-Pentixather showed higher accumulation in the nontargeted organs and tissues, such as lung, adrenals, spleen, and blood, while most importantly, it showed a very high liver uptake (10.3 ± 0.8% IA/g) at 1 h p.i., which remained unchanged over a period of 48 h (8.25 ± 2.23%IA/g).^17^ The authors reported part of this uptake to be CXCR4-mediated, which is contrary to the lower affinity of ^177^Lu-Pentixather to the murine CXCR4. Liver uptake is a significant drawback in radio-theragnostics given that (a) usually the washout is very slow and therefore radiation burden is high and (b) liver is a common site of metastasis of many cancers. Therefore, a low background activity is needed for good image contrast. Overall, a low background is important for diagnostic accuracy and for specific delivery of the radiation dose to the tumors, sparing healthy tissue. Recently, optimized derivatives of ^177^Lu-Pentixather were developed (*e.g*., [^177^Lu]DOTA-r-a-ABA-CPCR4) focusing on improving the ligand–receptor interaction,^41^ which indeed resulted in an increased tumor uptake. However, liver accumulation remained very high (11.9 ± 1.57%IA/g at 1 h p.i.) and compared to ^177^Lu-Pentixather, kidney uptake was significantly increased. In general, the kidney uptake of ^177^Lu-**7** is higher than for the radioligands reported in the literature, and we are currently working on different chemical approaches tackling this issue without compromising tumor uptake.

The total body distribution of ^68^Ga-**7** shared similar characteristics with ^68^Ga-Pentixafor,^42^ with the exception of the uptake in the kidneys being significantly higher for ^68^Ga-**7** than for ^68^Ga-Pentixafor (75.6 ± 30.3 *vs* 3.06 ± 0.63%IA/g, respectively). The activity in the blood was half for ^68^Ga-**7** than for ^68^Ga-Pentixafor at 1 h p.i. (0.52 ± 0.14 *vs* 1.08 ± 0.27%IA/g, respectively), indicating that ^68^Ga-**7** is eliminated faster from the bloodstream.

The specificity of ^177^Lu-**7** was assessed *in vivo* in the presence of the competitor AMD3100 in high excess. The tumor uptake of ^177^Lu-**7** was reduced by more than 60% (2.25 *vs* 0.87%IA/g), while reduction was observed also in other organs reported to naturally express CXCR4, such as the liver (5.19 *vs* 0.89%IA/g) and the spleen (0.81 *vs* 0.26%IA/g). As mentioned above, there is no direct evidence that ligand-**7** binds the murine isoform of CXCR4. However, the presence of AMD3100 significantly impacted in its uptake in the liver and spleen. This was not the case for radioligands that have limited to no cross-reactivity with mCXCR4, like ^68^Ga-Pentixafor^42^ or the radioligands derived from the N-terminal region of CXCRL12.^21^ A striking finding in our study was the reduction of the kidney uptake of ^177^Lu-**7** (83 *vs* 22%IA/g), which would suggest CXCR4 expression in the kidneys. To test this hypothesis, we attempted to stain mouse kidneys for mCXCR4. Formalin-fixed, paraffin-embedded kidneys from wild-type and nude mice were sectioned and stained with anti-mouse CXCR4. Alkaline phosphatase- and AF568-coupled secondary antibodies were used for detection. No specific staining of the kidney tissue was observed, suggesting that CXCR4 is indeed not (or only marginally) expressed. In addition, there is no evidence in the literature for CXCR4 expression in the kidneys. Similar to what was discussed above, the kidney uptake of other CXCR4-targeting radioligands (*e.g*., based on the CPCR4 scaffold,^16,17,41^ or on the peptide scaffold from the N-terminal region of CXCRL12^21^) was not impacted by the presence of a competitor. This finding deserves further investigation to conclude if this is due to an unknown binding to an off-target, or if it is specifically linked to the EPI-X4 scaffold.

## Conclusions

Our data support the feasibility of developing CXCR4-targeting radioligands related to the EPI-X4 scaffold, and especially based on its more active derivative JM#21. Stabilization strategies on both ends, N- and C-terminus, seemed to be crucial for *in vivo* total body residence time, while truncated derivatives might have an advantage (ligand-7). The *in vitro* and *in vivo* data of this first-generation radioligands based on JM#21 and its smaller versions designated ^177^Lu-**7** as the lead candidate for further optimization. The biodistribution of ^177^Lu-**7** has certain advantages compared to advanced CXCR4 radioligands. Further optimization is directed toward improvements related to CXCR4 affinity and metabolic stability.

## Materials and Methods

AMD3100 was purchased from Toronto Research Chemicals (Canada). All other reagents and solvents were purchased from Acros Organics (Belgium) and Merck (Darmstadt, Germany) and used without further purification. DOTA-tris(tBu)ester was purchased from CheMatech (France). Purity of the peptides was >95% as assessed by liquid chromatography mass spectrometry (LC-MS) on a LCMS-2020 SHIMADZU (Japan) system equipped with a Waters XBridge C-18 column (4.6 mm × 150 mm, 5 μm particle size). The gradient used was 15–65% solvent B in 15 min (*A* = H_2_O [0.1%TFA], *B* = ACN [0.1% TFA]) at a flow rate of 1.0 mL/min. Radio-HPLC was performed on an Agilent 1260 infinity instrument (Agilent) connected to a GABI radioactivity-HPLC-flow-monitor γ-spectrometer (Elysia-raytest, Germany). Radioligands were analyzed using Phenomenex Jupiter Proteo C12 (90 Å, 250 mm × 4.6 mm) column using the gradient 5–50% *B* in 15 min (*A* = H_2_O [0.1%TFA], *B* = ACN [0.1% TFA]) with a flow rate of 2 mL/min. Quantitative γ-counting was carried out on a COBRA 5003 γ-system well counter from Packard Instruments. SPECT/CT images were acquired using a dedicated nanoSPECT/CT system (Bioscan, Mediso, Hungary).

### Peptide Synthesis

All chemicals were used as provided by the manufacturers. Amino acids were purchased from Novabiochem (Merck KGaA, Darmstadt, Germany). *N*,*N*-Dimethylformamide (DMF), 20% (v/v) piperidine in DMF, *O*-benzotriazole-*N*,*N*,*N*′,*N*′-tetramethyluronium-hexafluoro-phosphate (HBTU), and trifluoroacetic acid (TFA) were purchased from Merck Millipore (Merck KGaA). Triisopropylsilane (TIS) and diisopropylethylamine (DIEA) were purchased from Merck (Darmstadt, Germany). Acetonitrile was purchased from JT. Baker (Avantor Performance Materials B.V. 7418 AM Deventer Netherlands). The peptides were synthesized automatically on a 0.10 mmol scale using standard Fmoc solid phase peptide synthesis techniques with the microwave synthesizer (Liberty blue; CEM). A preloaded resin was used and provided in the reactor. The resin was washed with DMF. The Fmoc protecting group was removed with 20% (*v*/*v*) piperidine in DMF and initialized with microwave followed by washing with DMF. Amino acids were added in 0.2 mol/L equivalent to the reactor, and then HBTU in a 0.5 mol/L equivalent was dosed to the amino acid solution. After that, 2 mol/L equivalent of DIEA was added to the resin. The coupling reaction was proceeded with microwaves for a few minutes and then the resin was washed in DMF. These steps were repeated for all amino acids in the sequence. Following the addition of the last amino acid, Fmoc was deprotected and DOTA-tris(tBu)ester (0.2 mol/L) was added to the reaction mixture. Once the synthesis was completed, the peptide was cleaved in 95% (v/v) TFA, 2.5% (v/v) TIS, and 2.5% (v/v) H_2_O for 1 h. The peptide residue was precipitated and washed with cold diethyl ether (DEE) by centrifugation. The peptide precipitate was then allowed to dry under air flow to remove residual ether. The peptide was purified using RP-HPLC (Waters) in an acetonitrile/water gradient under acidic conditions on a Phenomenex C-18 Luna column (5 mm pore size, 100 Å particle size, 250 mm × 21.2 mm). Following purification, the peptide was lyophilized on a freeze dryer (Labconco) for storage prior to use. The purified peptide mass was verified by LC/MS (Shimadzu) by injecting 10 μL from an aliquot of 1 mg/mL solution using the gradient 15–65% solvent B in 15 min (*A* = H_2_O [0.1%TFA], *B* = ACN [0.1% TFA]).

### Complexes of the Ligands with Natural Lutetium

All ligands were complexed with ^nat^Lu. For this purpose, 1 mg was dissolved in 250 μL of ammonium acetate buffer (0.4 M, pH 5.2), followed by the addition of 2.5-fold excess of ^nat^LuCl_3_ × 6H_2_O (1 M) and incubated at 95 °C for 30 min. After incubation, ^nat^Lu ligands were separated from free metal ions by a SepPak C-18 cartridge, preconditioned with methanol (10 mL) and Milli-Q-water (10 mL). The reaction mixture was loaded and the free ^nat^Lu was eluted with Milli-Q-water (10 mL), while the ^nat^Lu ligands were eluted with methanol (10 mL). The methanol phase was later evaporated on a Rotavapor (Büchi, Switzerland), redissolved in water (2 mL), and lyophilized. The cysteine-bearing peptides that exhibited two peaks after complexation were separated by semipreparative RP-HPLC and characterized by MALDI-TOF (Shimadzu 8020, Japan). Other ^nat^Lu ligands were characterized by LC-MS for determining their purity and mass.

### ^177^Lu Labeling

^177^Lu-labeled ligands were synthesized by dissolving 5–10 μg (3–6 nmol) of the ligand in 250 μL of ammonium acetate buffer (0.4 M, pH 5.0) followed by incubation with ^177^LuCl_3_ (100–400 MBq, depending on the planned experiment). Labeling of cysteine-free peptides (2, 5, 7, 9) was performed in the presence of 10% ethanol at 95 °C for 30 min. The stock solutions of cysteine-bearing peptides (1, 6, 8) were prepared in dithiothreitol (DTT, 10 mM final concentration) and radiolabeling was also performed in the presence of 10% ethanol at 75 °C for 30 min. For quality control, an aliquot of 5 μL was withdrawn from the mother solution and diluted in 50 μL of calcium diethylenetriamine pentaacetate (Ca-DTPA) solution. Ca-DTPA was used to quench the reaction and complex any unreacted ^177^Lu^3+^, in the form of ^177^Lu-DTPA. 20 μL of this solution was injected into a radio-HPLC system using the gradient mentioned above. The radioligands were diluted with 0.9% NaCl containing 0.05% human serum albumin to a final concentration of 1 μM (stock solution).

### ^68^Ga Labeling

Ligand-**7** (3–5 nmol) was labeled with [^68^Ga]GaCl_3_, retrieved from a ^68^Ge/^68^Ga-generator (IGG100, Eckert & Ziegler, Berlin, Germany) at an apparent molar activity of 50 MBq/nmol. Labelings were performed in sodium acetate buffer (0.2 M, pH 4.2, supplemented with 10% ethanol) at 95 °C for 15 min. Quality control and the final dilutions were done as described above for the ^177^Lu-labeled ligands.

### Shelf-Life

The shelf-lives of ^177^Lu-labeled ligands were assessed at room temperature (RT) in the buffer used for labeling, as described above. For the analysis, 5 μL was withdrawn at desired time points (0, 1, 2, 4, and 24 h) from the stock solution and mixed with 50 μL of the Ca-DTPA solution. 20 μL of this mixture was withdrawn and injected in RP-HPLC.

### Log *D* Determination

Log* D*_(pH = 7.4)_ determination of the ^177^Lu ligands was performed by the shake-flask method. In a prelubricated eppendorf tube, a presaturated mixture of 500 μL of octanol and 500 μL of phosphate-buffered saline (PBS) were added. 1 pmol of the ^177^Lu-labeled ligand was pipetted into this mixture, vortexed for 30 min, and then centrifuged at 3000 rpm for 10 min to achieve phase separation. Aliquots of 100 μL were removed from the octanol and from the PBS phase and the activity measured in a γ-counter. The distribution coefficient was calculated as the average log ratio value of the radioactivity in the organic fraction and PBS fraction.

### *In Vitro* Metabolic Stability and Plasma Protein Binding

To 1 mL of fresh human plasma, previously equilibrated at 37 °C, 500 pmol (8–10 MBq) of each ^177^Lu-labeled ligand was added. The mixture was incubated at 37 °C for different time points (0, 15, 30, 60, and 120 min). At each time point, a 100 μL aliquot was removed and treated with 1.5 excess (v/v) of acetonitrile, to precipitate plasma proteins, followed by centrifugation for 5 min at 3500 rpm. The supernatant was collected and the process of protein precipitation was repeated. Afterward, 100 μL from the supernatant was taken, diluted with water at a ratio of 1:10 (v/v), and injected into radio-HPLC. The radioligand (in)stability was assessed by quantifying the percentage of intact radioligand over time.

For the determination of plasma protein binding (PPB), 100 μL from the plasma incubated with the ^177^Lu ligand was removed and measured in a γ-counter (total radioactivity). Protein precipitation and separation followed, as described above. The supernatant and the protein pellet were then measured in a γ-counter. The percentage of plasma protein binding was calculated as the ratio between the protein-bound radioactivity and the total radioactivity in the plasma.

### Cell Lines

Ghost-CXCR4 is a human osteosarcoma cell line stably expressing CD4 and CXCR4, and it was obtained from the NIH HIV Reagent Program (see acknowledgments). The Jurkat cell line, clone E6-1, is derived from human T lymphoblasts and it was obtained from ATCC. Ghost cells stably transfected with CXCR4 (Ghost-CXCR4) were cultured in Dulbecco’s Modified Eagle Medium (DMEM) supplemented with 10% fetal bovine serum (FBS), 100 units/mL penicillin, 100 μg/mL streptomycin, 2 mmol/mL l-glutamine, 1 μg/mL puromycin (BioConcept), 100 μg/mL hygromycin (BioConcept), and 500 μg/mL geneticin G418 (BioConcept). Jurkat cells were grown as a suspension in RPMI-1640 supplemented with 10% FBS (Merck), 100 units/mL penicillin, 100 μg/mL streptomycin (BioConcept), and 2 mmol/mL l-glutamine (Gibco).

### Antibody-Competition Assay

Competition of ligands with antibody binding was performed on Ghost-CXCR4 and Jurkat cells according to a previously established protocol.^26^ In brief, cells were washed in PBS containing 1% FBS and were then seeded in a 96 V-well plate (50,000 cells/well). The buffer was removed and plates were precooled at 4 °C. Ligands were serially diluted in PBS. The antibody (clone 12G5, Allophycocyanin labeled, BD Pharmingen #555976) was diluted in PBS containing 1% FBS. The antibody was used at a fixed concentration close to its *K*_D_. 15 μL of compound and immediately afterward 15 μL of the antibody were added to the cells. Plates were incubated at 4 °C in the dark for 2 h allowing to establish an equilibrium binding. Cells were then washed twice with PBS containing 1% FBS and fixed with 2% paraformaldehyde. Antibody binding was analyzed by flow cytometry (FACS CytoFLEX; Beckman Coulter).

### Competition Binding Assay with [^125^I]SDF-1α

The affinities of the ^nat^Lu-labeled ligands for CXCR4 were determined using a cell-based competition binding assay. Jurkat cells in suspension (400,000) were incubated with [^125^I]SDF-1α (0.01 nM, PerkinElmer), following by the addition of increased concentrations of ^nat^Lu ligands (0.001–10 μM). Nonspecific binding was determined in the presence of AMD3100 (100 μM). The mixture was incubated for 1 h on a thermomixer at 37 °C with moderate shaking. Following incubation, the mixture was centrifuged at 14,000 rpm for 5 min. Next, the supernatant was removed and the cell pellets were washed once with cold PBS followed by a second centrifugation step. Finally the cell-bound radioactivity was counted in a γ-counter. Experiments were repeated 2–3 times in triplicates. IC_50_ values were determined by a nonlinear regression analysis using GraphPad Prism 9.

### Cellular Uptake

The receptor-specific cell surface-bound and internalization kinetics of the ^177^Lu ligands were studied in Ghost-CXCR4 cells (10^5^ cells/well) seeded in a 24-well plate, as described in the literature.^41^ Briefly, the cells were preconditioned in DMEM supplemented with 5% bovine serum albumin (BSA) at 37 °C for 30 min. ^177^Lu ligand (1 nM) was added and the cells were incubated at 37 °C. Cellular uptake was interrupted at 15, 30, and 60 min by removing the medium and washing twice with ice-cold PBS. The cell surface-bound radioligand was obtained by washing cells twice with ice-cold glycine buffer (pH 2.8) followed by a collection of the internalized fraction after treating the cells with 1 M sodium hydroxide (NaOH). The activity in each fraction was measured in a γ-counter. Nonspecific binding was determined in the presence of 100,000-fold excess of AMD3100. The results were expressed as a percentage of the applied radioactivity after subtracting the nonspecific uptake.

### Molecular Modeling

The previously reported model of the JM#173/CXCR4 complex was employed as the starting structure,^27^ where the structure of CXCR4 reported by Sokkar et al.^24^ was used. More precisely, Sokkar et al. built a model of CXCR4 by combining the crystal structure of the transmembrane domain (PDB ID: 3ODU)^43^ of CXCR4 and the NMR structure of the N-terminal region (PDB ID: 2K04).^44^ We modified the JM#173 peptide bound to CXCR4 with the addition of an amidated C-terminal Lys. Then, the Nε atom of the Lys side chain was linked to the DOTA group, using the DOTA structure (without the Gd atom) reported for the ligand DOF in the PDB file with ID 1NC4.^37^ One of the carboxylic groups of DOF was modified to form an amide bond with the Nε atom of the Lys side chain.

The modified peptide bound to CXCR4 was subjected to geometry optimization with the Sculpting tool in Pymol (The PyMOL Molecular Graphics System, V.S., LLC), to eliminate clashes and correct unfavorable angles. To explore other binding possibilities, the modified peptide was subsequently extracted and docked again into the binding site of CXCR4, this time with the HADDOCK 2.4 webserver,^45,46^ using the Ligand protocol modified with the parameters of the Peptide protocol. Since the HADDOCK tool does not support it, this run was performed without the Lu atom, which was subsequently added. From the resulting docking poses, those with the modified peptide in the binding site were selected. The Lu^3+^ ion was placed at the center of the DOTA group by replacing the Gd atom in the structure of DOTA reported in the PDB file with ID 1NC4. Subsequently, the electronic density map of the DOTA group (from PDB ID 1NC4) was used to refine the structure of the group to coordinate the Lu^3+^ ion. A final round of energy minimization with the Pymol’s Sculpting tool of the binding interface was performed as a refinement.

### Jurkat Xenografted Mouse Model for *In Vivo* Studies

The experimental protocol involving animals was approved by the Veterinary Office (Department of Health) of the Cantonal Basel-Stadt (Approval No. 30515) in accordance with the Swiss regulations for animal treatment. Female athymic nude-*Foxn*1^nu^/*Foxn*1^+^ mice (Envigo, The Netherlands), 4–6 weeks old, were injected subcutaneously with 10^7^ Jurkat cells in a 200 μL (1:1) mixture of PBS and Matrigel (CorningMatrigelMembrane matrix, Fischer Scientific, Germany) on the right shoulder. The tumors were allowed to grow for 3–4 weeks before commencement of the experiments.

### Total Body SPECT/CT and PET/CT Imaging

Nude mice bearing Jurkat xenografts were intravenously injected *via* the tail vein with ∼15 MBq (200 pmol) of the ^177^Lu ligands and 8 MBq (200 pmol) of ^68^Ga-**7** were euthanized 1 h post injection (p.i.). The radioactivity in the syringe before and after injection was measured followed by measuring the amount of radioactivity remaining in the sacrificed mice. The mice were imaged supine, head first, using a SPECT/CT system (NanoSPECT/CT Bioscan Inc.). Topograms and helical CT scans of the whole mouse were first acquired using the following parameters: X-ray tube current 177 mA, X-ray tube voltage 45 kVp, 90 s and 180 frames per rotation, pitch 1. CT images were reconstructed using CTReco (version r1.146), with a standard filtered back projection algorithm (exact cone beam) and post-filtered (RamLak, 100% frequency cut-off), resulting in a pixel size of 0.2 mm.

A helical SPECT scan of the ^177^Lu ligands was acquired using multipurpose pinhole collimators (APT1), 20% energy window width centered symmetrically over the 208 and 113 keV g-peaks of ^177^Lu, 24 projections, and 1000 s per projection. SPECT images were reconstructed iteratively and filtered using the HiSPECT software package (version 1.4.1876, SciVis GmbH, Goettingen, Germany) and the manufacturer’s algorithm (three subsets, nine iterations, 35% post-filtering, 128 × 128 matrix, zoom 1, 30 mm × 20 mm transaxial field of view, resulting in a pixel size of 0.3 mm). Coregistered SPECT/CT images were visualized in the three orthogonal planes using maximum intensity projection (MIP) with InVivoScope (version 1.43, Bioscan Inc.).

The PET images of ^68^Ga-**7** were acquired in list mode using a small animal PET scanner (β-cube, Molecubes, Ghent, Belgium) with a spatial resolution of 0.85 mm and an axial field of view of 13 cm. Static PET scan was acquired at 60 min, decay corrected, and reconstructed into a 192 × 192 × 384 matrix by an ordered subsets maximization expectation (OSEM) algorithm using 30 iterations, a voxel size of 400 μm × 400 μm × 400 μm. CT data was used to apply attenuation correction on the PET data. Coregistered PET/CT images were visualized using maximum intensity projection (MIP) with VivoQuant software (version 4.0.).

### Biodistribution Studies

Quantitative biodistribution studies of ^177^Lu-**7** (100 μL, 200 pmol, 0.8–1 MBq) and ^68^Ga-**7** (100 μL, 200 pmol, 2–3 MBq) were performed after intravenous injection of the radioligand *via* the tail vein on Jurkat xenografts. Blocking studies to assess specificity were performed in the presence of AMD3100 (60 nmol). 1 h after injection of the radioligand, mice were sacrificed, organs of interest and blood were collected, rinsed of excess blood, blotted dry, weighed, and counted in a γ-counter. The samples were counted against a suitable diluted aliquot of the injected solution as the standard and the results are expressed as percentage injected activity per gram of tissue (%IA/g). Statistical analysis between selected organs was performed by Welch’s *t*-test and *p* values <0.05 were considered statistically significant.

## Supporting Information Available

UV chromatograms and MS spectra of all of the ligands; chromatograms of ^nat^Lu ligands; MALDI-TOF of ^nat^Lu-1 and ^nat^Lu-6; radiochromatogram of ^177^Lu-8; antibody-competition assay on Ghost-CXCR4 and Jurkat cells; correlation analysis of IC_50_ between Ghost-CXCR4 and Jurkat cells; *in vitro* metabolic stability chromatograms of ^177^Lu-**1**, ^177^Lu-**2**, and ^177^Lu-**7**, and SPECT/CT image of ^177^Lu-**3** in Jurkat xenograft (PDF)
CXCR4_JM173-DOTA-Lu3 (PDB)

## Supplementary Material

jm3c00131_si_001.pdf

jm3c00131_si_002.pdb

## Abbreviations Used

ACN: acetonitrile
BSA: bovine serum albumin
ca-DTPA: calcium diethylenetriamine pentaacetate
CXCR4: C–X–C chemokine receptor 4
DIEA: diisopropylethylamine
DMEM: Dulbecco’s Modified Eagle’s Medium
DMF: *N*,*N*-dimethylformamide
DOTA: 1,4,7,10-tetraazacyclododecane-1,4,7,10-tetraacetic acid
DTT: dithiothreitol
EPI-X4: endogenous peptide inhibitor of CXCR4
FBS: fetal bovine serum
HBTU: *O*-benzotriazole-*N*,*N*,*N*′,*N*′-tetramethyluronium-hexafluoro-phosphate
%IA/g: percentage injected activity per gram of tissue
LC-MS: liquid chromatography mass spectrometry
MALDI-TOF: matrix-assisted laser desorption ionization time of flight
NaOH: sodium hydroxide
nM: nanomolar
^nat^Lu: natural lutetium
PBS: phosphate-buffered saline
PET: positron emission tomography
p.i.: post injection
RT: room temperature
RCP: radiochemical purity
RCY: radiochemical yield
RP-HPLC: reverse phase high-performance liquid chromatography
SPECT: single-photon emission computed tomography
TLC: thin layer chromatography
TFA: trifluoroacetic acid
TIS: triisopropylsilane
PDB: protein data bank
