# Supplementary material for: Development of a New Class of CXCR4-Targeting Radioligands Based on the Endogenous Antagonist EPI-X4 for Oncological Applications
Source: J Med Chem. 2023 Jun 16;66(13):8484–97. doi: 10.1021/acs.jmedchem.3c00131 (PMC10350915; doi:10.1021/acs.jmedchem.3c00131)
Supplement: Supplementary file 1 — jm3c00131_si_001.pdf [file jm3c00131_si_001.pdf]

Development of a new class of CXCR4 targeting radioligands based on the endogenous antagonist EPI-X4 for oncological applications

Raghuvir Haridas Gaonkar[1], Yannik Tim Schmidt[1], Rosalba Mansi[1], Yasser Almeida-Hernanadez[2,3], Elsa Sanchez-Garcia[2,3], Mirja Harms[4], Jan Münch[4,5], Melpomeni Fani[1]*

[1]Division of Radiopharmaceutical Chemistry, Department Theragnostics, University Hospital Basel, Basel 4031, Switzerland

[2]Computational Biochemistry, Center of Medical Biotechnology, University of Duisburg-Essen, Essen 45117, Germany

[3]Computational Bioengineering, Faculty of Bio- and Chemical Engineering, Technical University Dortmund, Dortmund 44227, Germany

[4]Institute of Molecular Virology, Ulm University Medical Center, Ulm 89081, Germany

[5]Core Facility Functional Peptidomics, Ulm University Medical Center, Ulm 89081, Germany

*Corresponding author. Tel.: +41 (0)61 556 58 91

E-mail: melpomeni.fani@usb.ch

Table of contents

LC-MS analytics of the ligands……………………..……………….…………..…… S3

LC-MS analytics of the ^{nat}Lu-complexed ligands…………………………………..............S3

UV-Chromatograms of the ligands…………………………………………….………........S4-S8

Antibody competition assay on Ghost-CXCR4 cells…………………………………………S9

Antibody competition assay on Jurkat cells…...……………………………………………..S10

Correlation analysis of IC_{50} between Ghost-CXCR4 and Jurkat cells………………………..S11

UV-Chromatograms of ^{nat}Lu-ligands…………………………………………...........S12-S18

MALDI-TOF of ^{nat}Lu-**1**……………………………………………………………...S13

MALDI-TOF of ^{nat}Lu-**6**……………………………………………………………...S16

Radiochromatogram of ^{177}Lu-**8**…………………………………………………………...S18

In vitro metabolic stability chromatograms of ^{177}Lu-**1**, ^{177}Lu-**2** and ^{177}Lu-**7**…………………S19

SPECT/CT image of ^{177}Lu-**3** in Jurkat xenograft……………………………………………S20

Table S1. LC-MS analytics of the ligands.

Ligand	Calculated $[M+2H]^{2+}$	Observed $[M+2H]^{2+}$	HPLC t_R (min)
**1**	987.19	987.12	10.1
**2**	979.16	979.12	9.8
**3**	842.12	842.41	10.9
**4**	736.88	736.91	9.8
**5**	672.80	672.80	8.9
**6**	987.19	987.14	11.1
**7**	736.89	736.85	9.9
**8**	1078.36	1078.30	10.4
**9**	920.65	920.60	19.1

Gradient: 15-65% solvent B in 15 min (*A* = H_2O [0.1%TFA], *B* = ACN [0.1% TFA])

Table S2. LC-MS analytics of the ^{nat}Lu-complexed ligands.

Ligand	Calculated $[M+2H]^{2+}$	Observed $[M+2H]^{2+}$	HPLC t_R (min)
^{nat}Lu-**1**	1074.68	1073.10	10.3
^{nat}Lu-**2**	1066.65	1065.04	9.7
^{nat}Lu-**5**	760.29	758.72	8.6
^{nat}Lu-**6**	1074.68	1073.05	11.2
^{nat}Lu-**7**	824.36	822.85	11.3
^{nat}Lu-**8***	1165.85	1164.29	15.4
^{nat}Lu-**9***	1008.14	1006.56	6.3

Gradient: 15-65% solvent B in 15 min (*A* = H_2O [0.1%TFA], *B* = ACN [0.1% TFA])
*30-70 % solvent B in 15 min (*A* = H_2O [0.1%TFA], *B* = ACN [0.1% TFA])

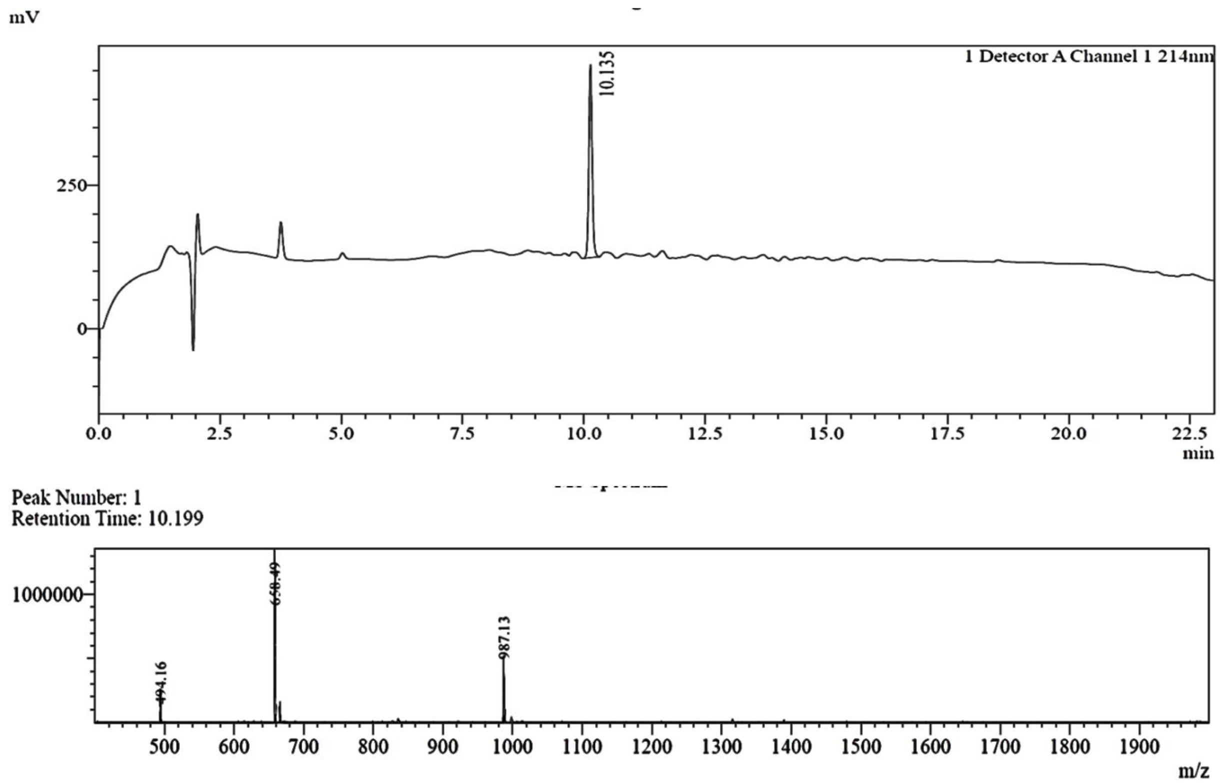

Figure S1. LC-MS profile of ligand-**1**

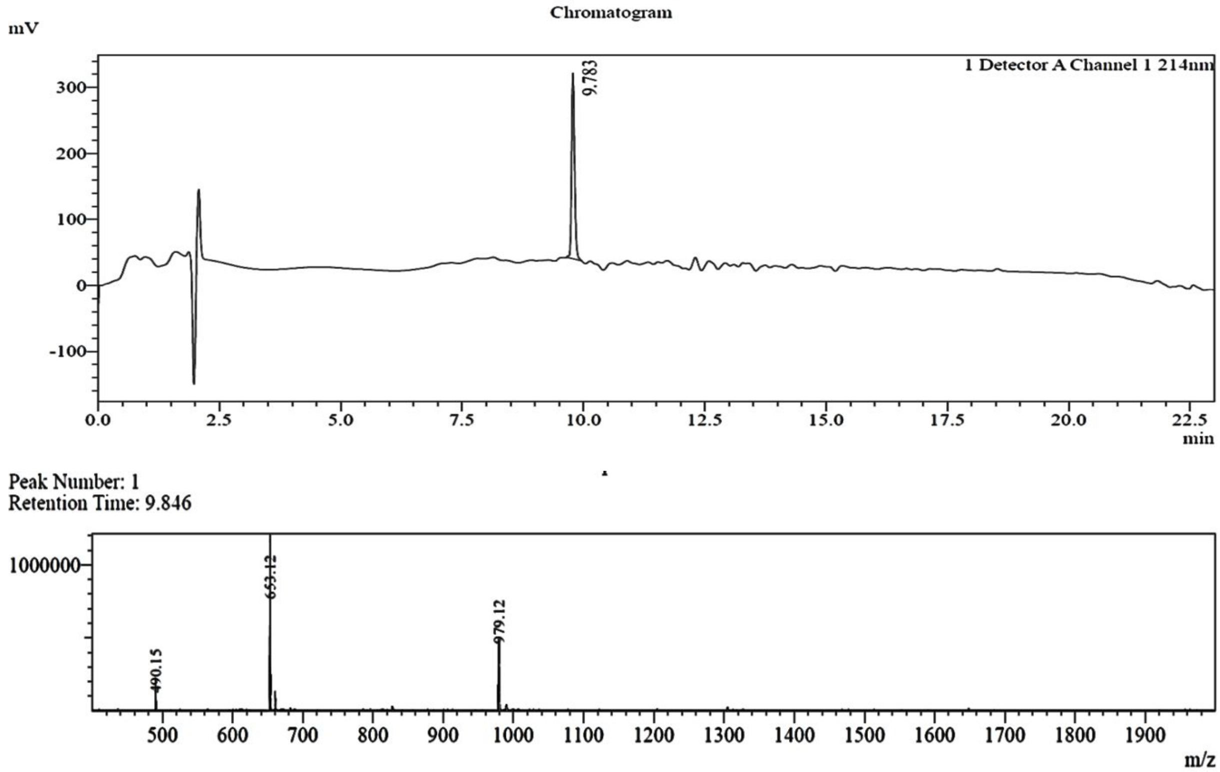

Figure S2. LC-MS profile of ligand-**2**

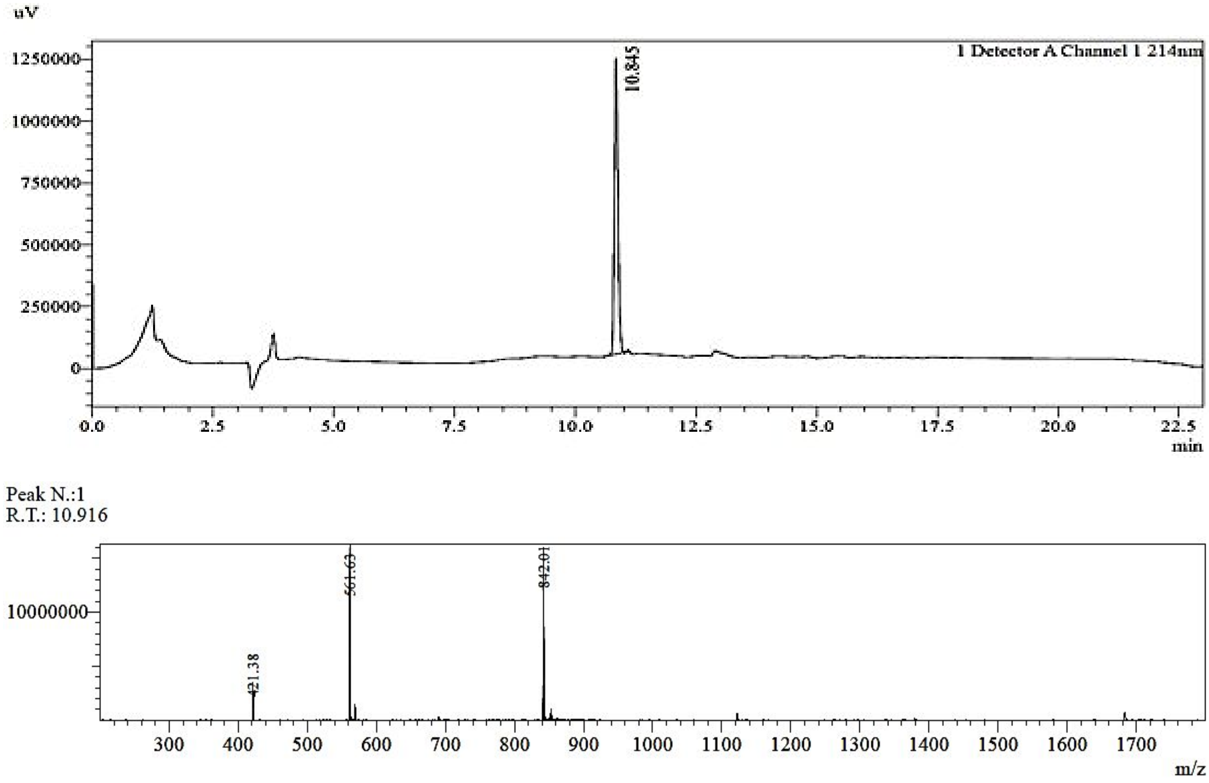

Figure S3. LC-MS profile of ligand-**3**

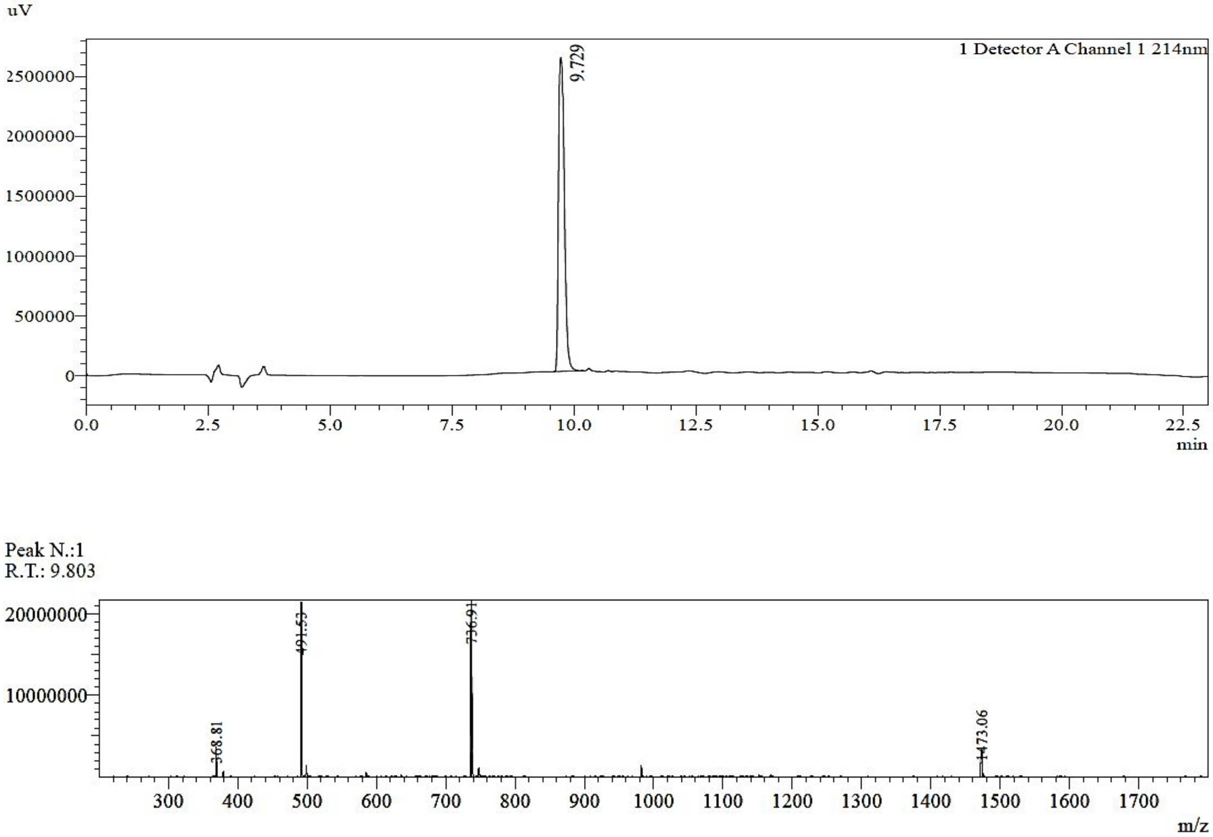

Figure S4. LC-MS profile of ligand-**4**

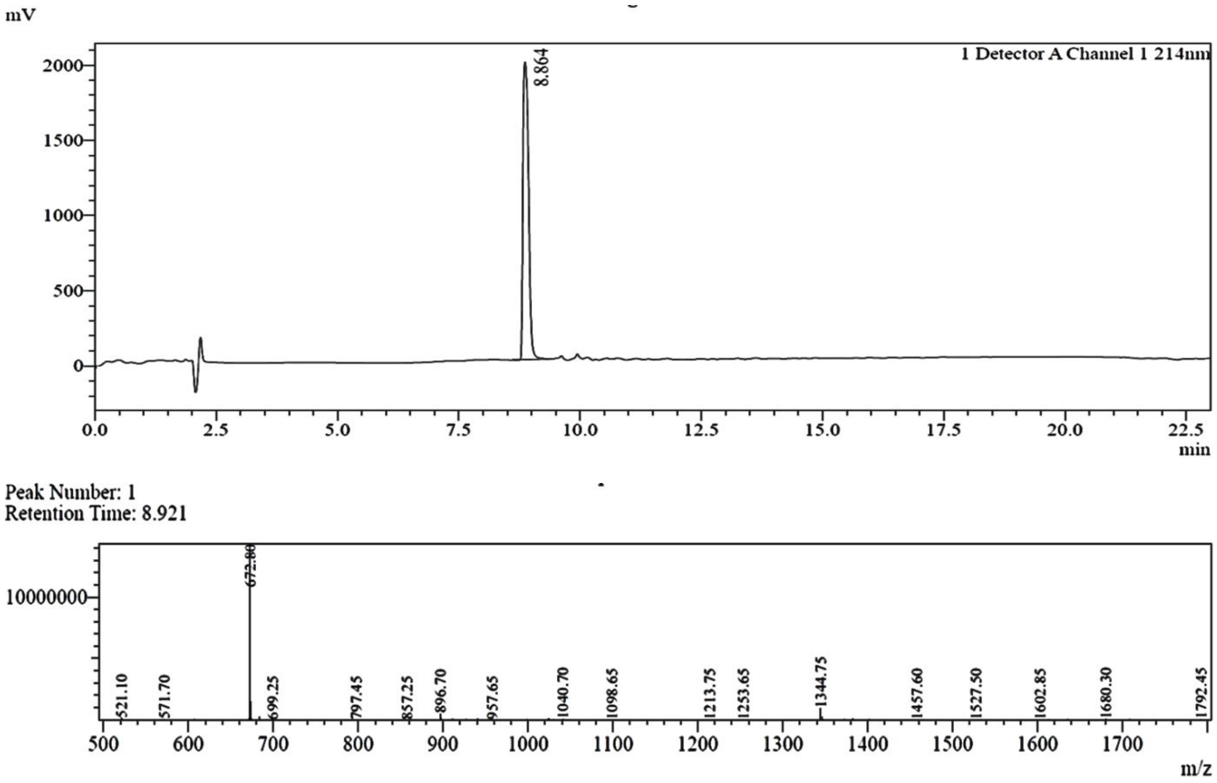

Figure S5. LC-MS profile of ligand-**5**

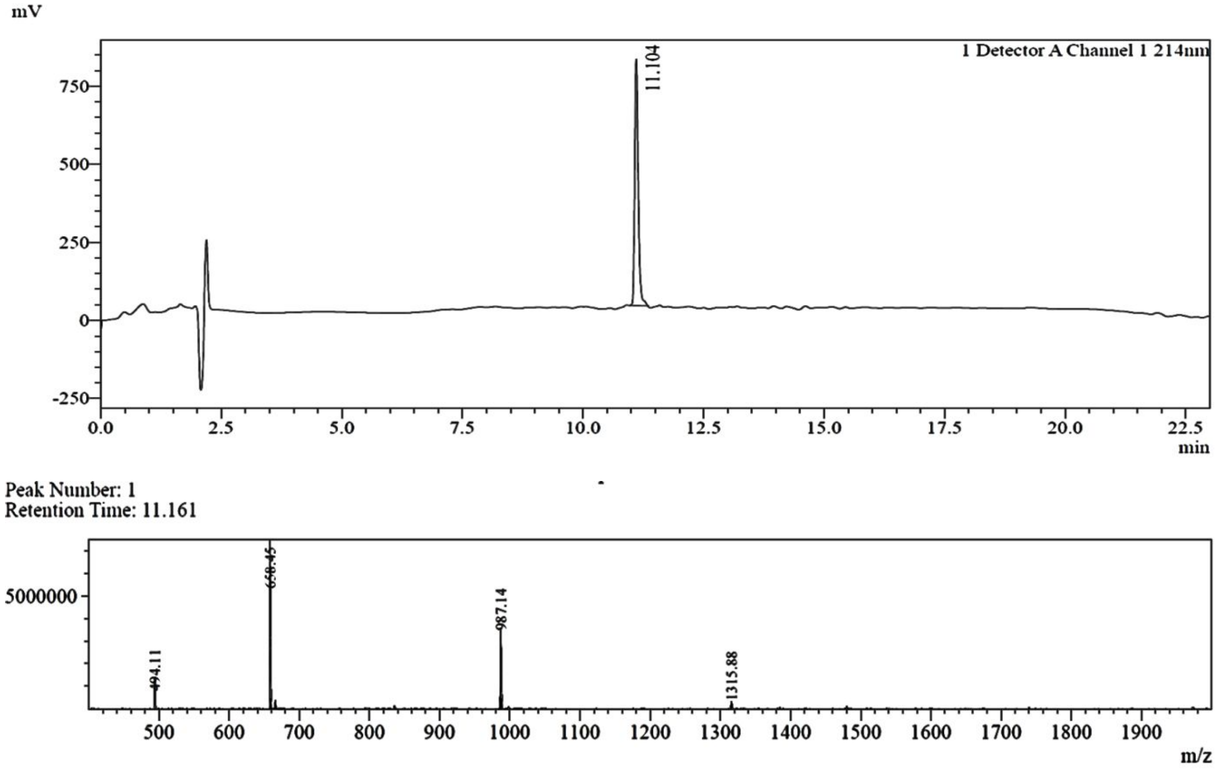

Figure S6. LC-MS profile of ligand-**6**

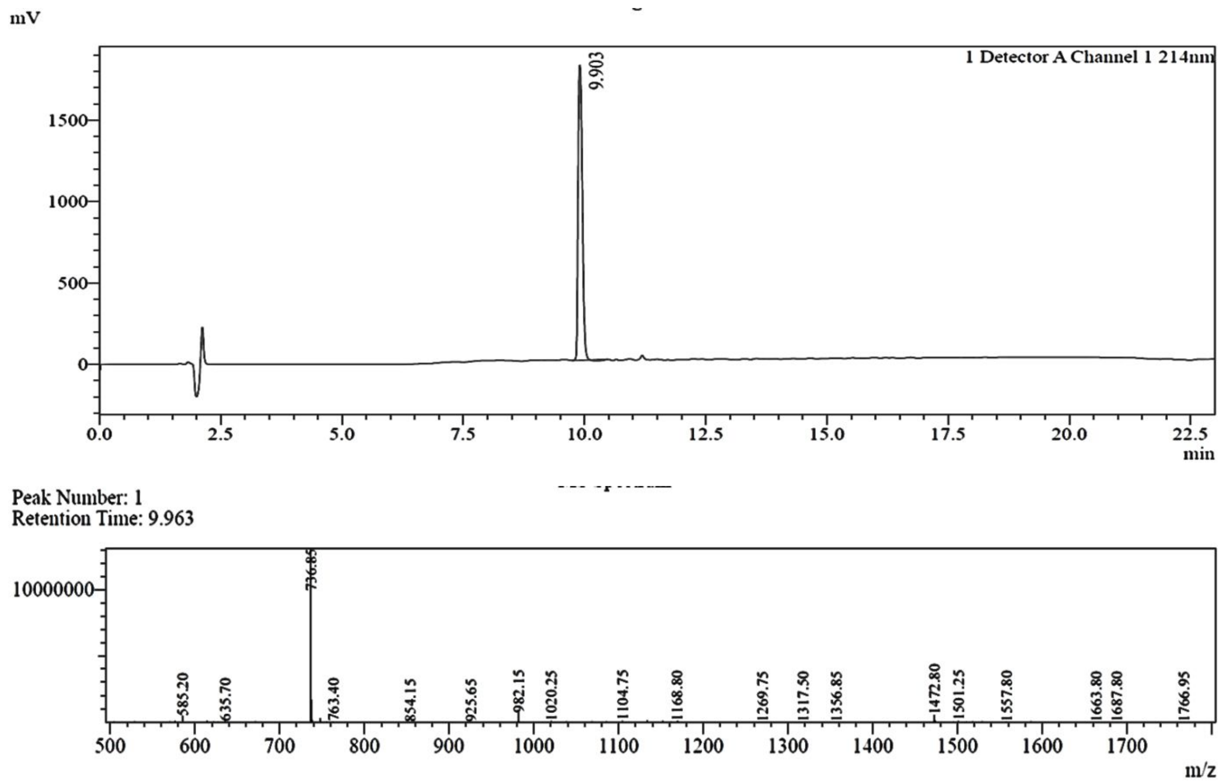

Figure S7. LC-MS profile of ligand-**7**

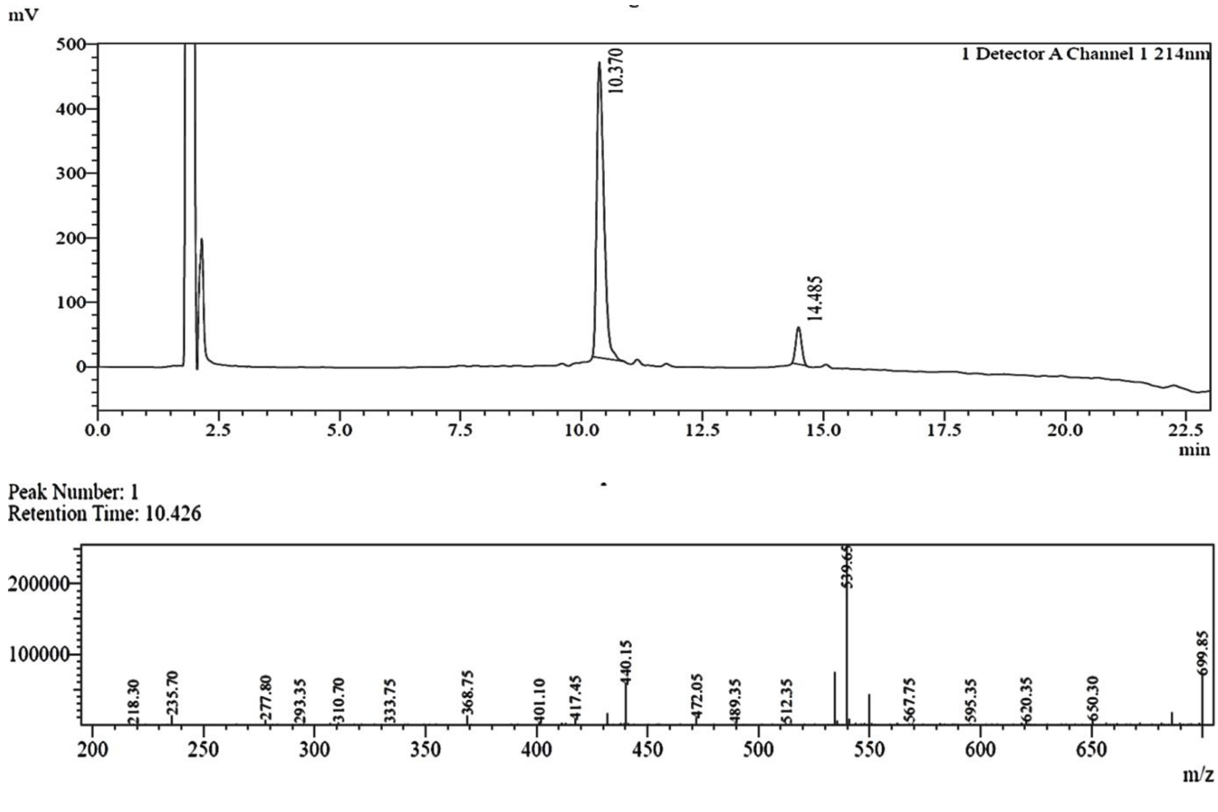

Figure S8. LC-MS profile of ligand-**8**

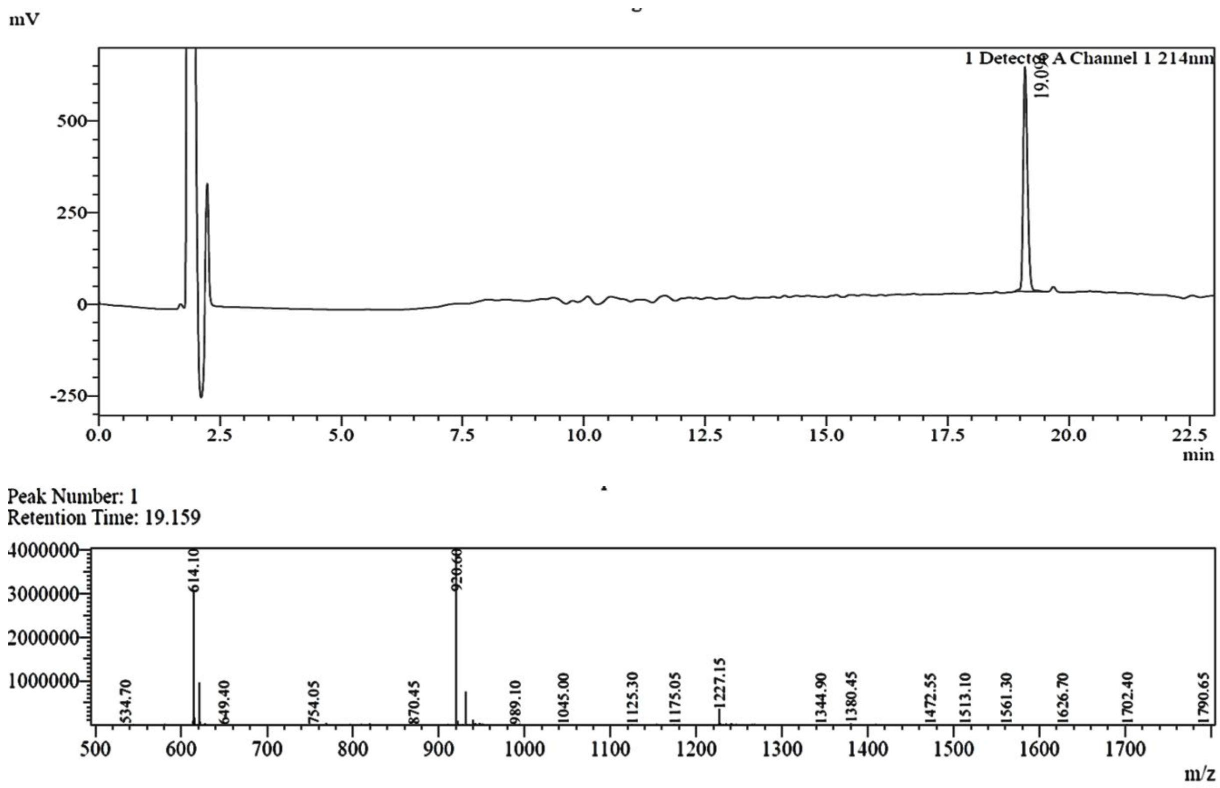

Figure S9. LC-MS profile of ligand-**9**

Ghost-CXCR4

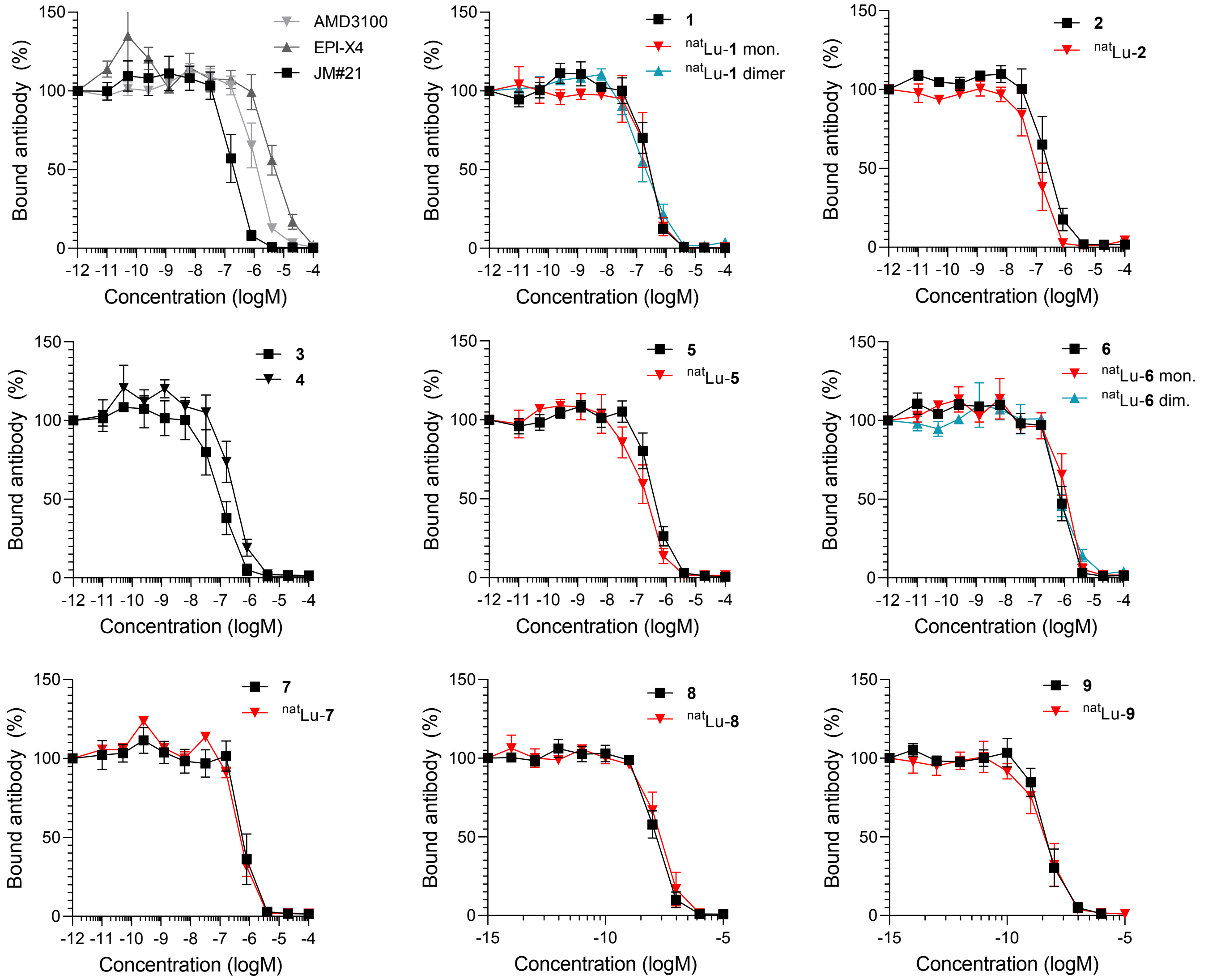

Figure S10. Ligands compete with a CXCR4 antibody on Jurkat cells. Ligands were serially diluted in PBS before they were added to 50’000 Jurkat cells together with a constant concentration of a CXCR4 antibody (clone 12G5). After 2 hours incubation at 4°C, unbound antibody was removed and cells analyzed by flow cytometry. Shown are means derived from 3-5 independent experiments ± SEM. IC_{50} values were determined by non-linear regression.

Jurkat

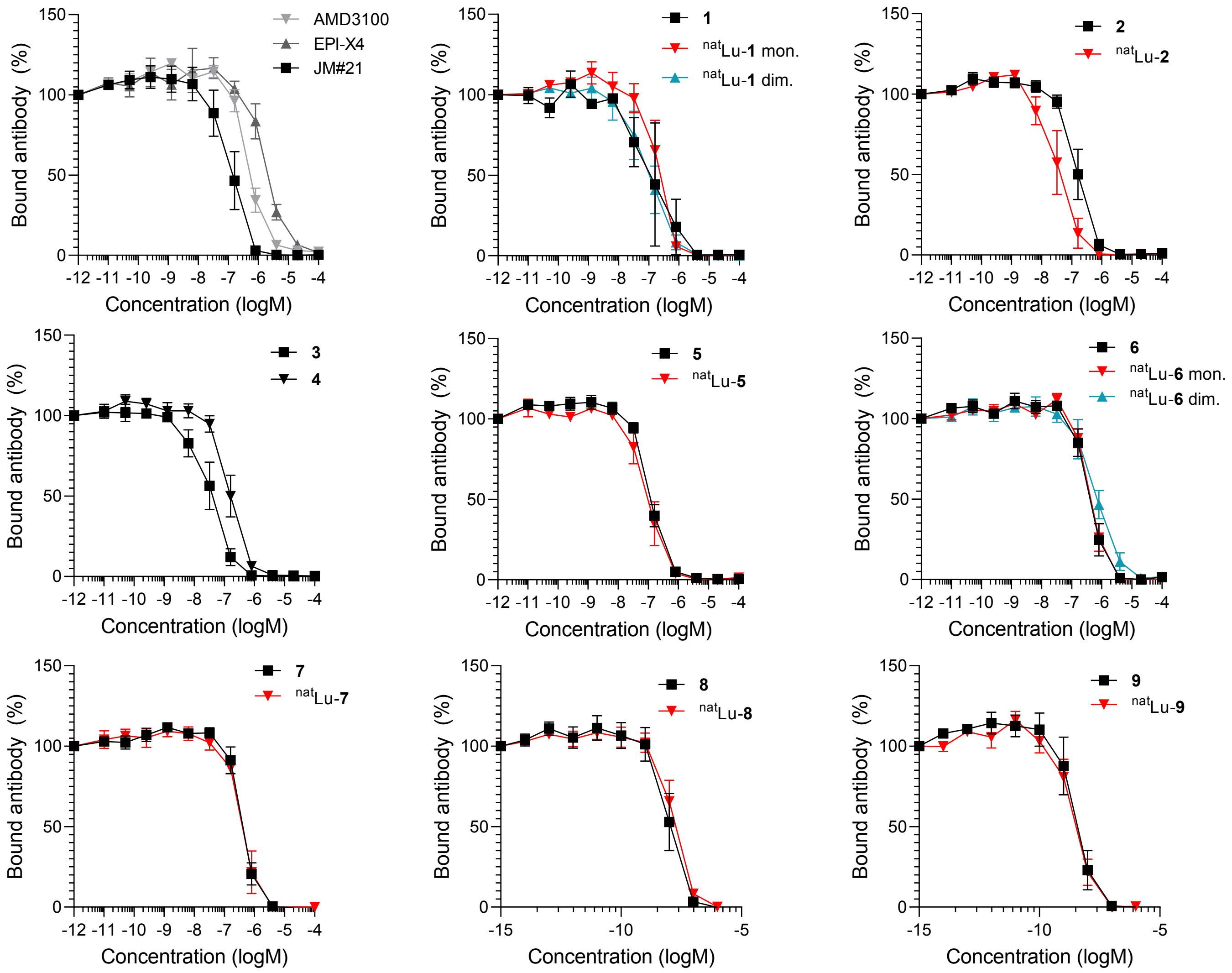

Figure S11. Ligands compete with a CXCR4 antibody on Jurkat cells**.** Ligands were serially diluted in PBS before they were added to 50'000 Jurkat cells together with a constant concentration of a CXCR4 antibody (clone 12G5). After 2 hours incubation at 4°C, unbound antibody was removed and cells analyzed by flow cytometry. Shown are means derived from 3-5 independent experiments ± SEM. IC_{50} values were determined by non-linear regression.

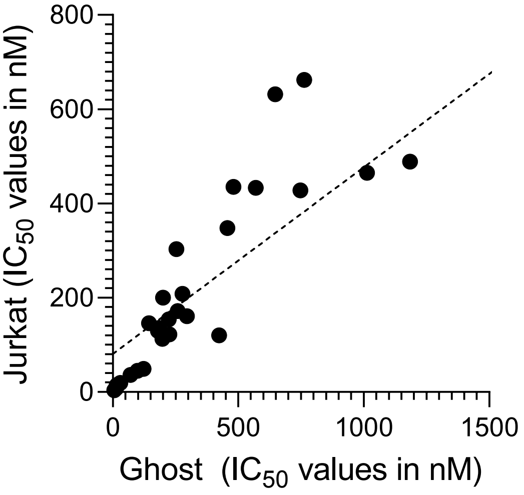

	Ghost vs. Jurkat
Pearson r	
r	0,9519
95% confidence interval	0,9005 to 0,9771
R squared	0,9062
P value	
P (two-tailed)	<0,0001
P value summary	****
Significant? (alpha = 0.05)	Yes
Number of XY Pairs	30

Figure S12. Pearson correlation analysis between the IC_{50} values in Ghost-CXCR4 and Jurkat cells indicating a strong correlation.

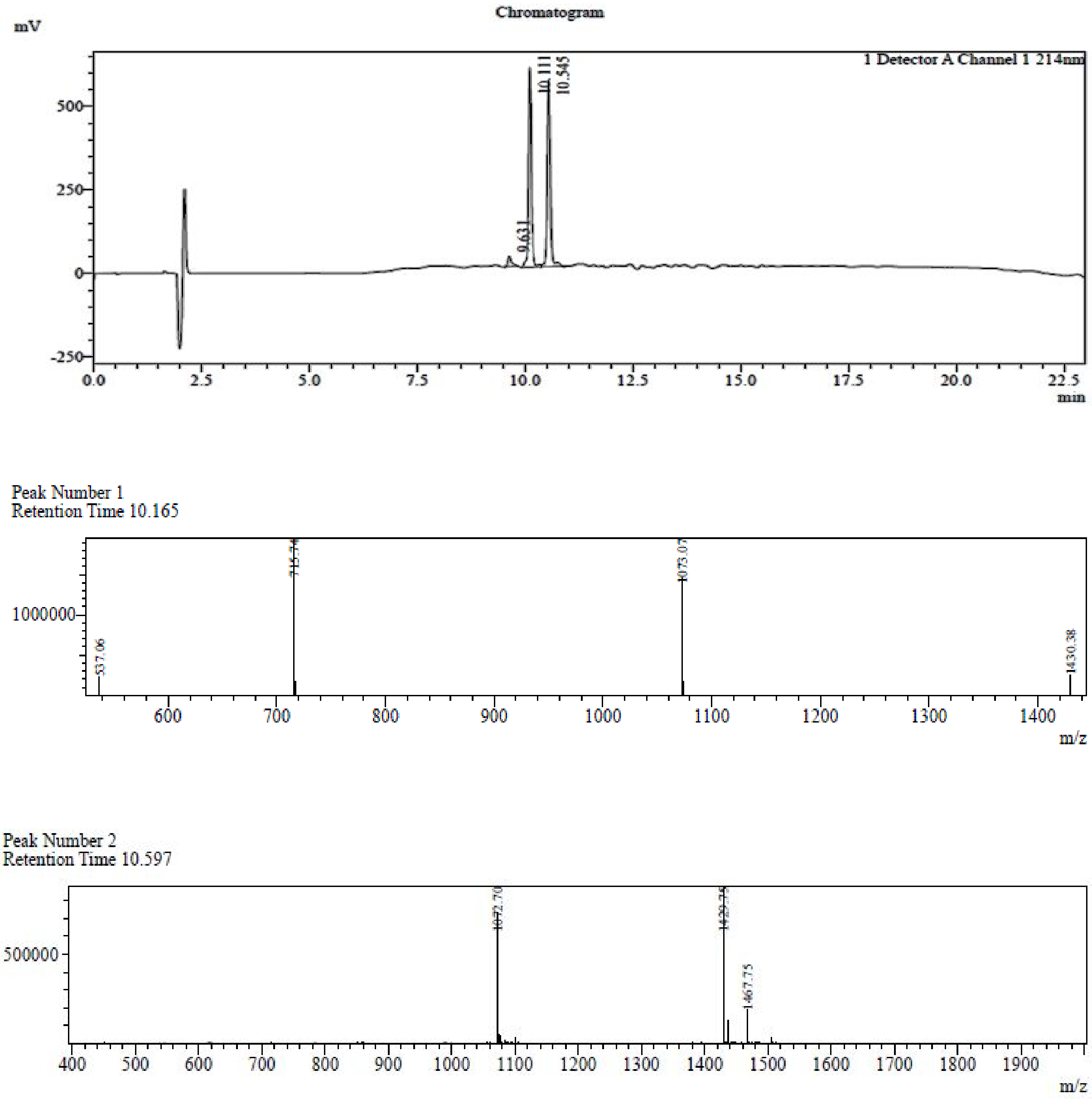

Figure S13. LC-MS profile of ^{nat}Lu-**1**

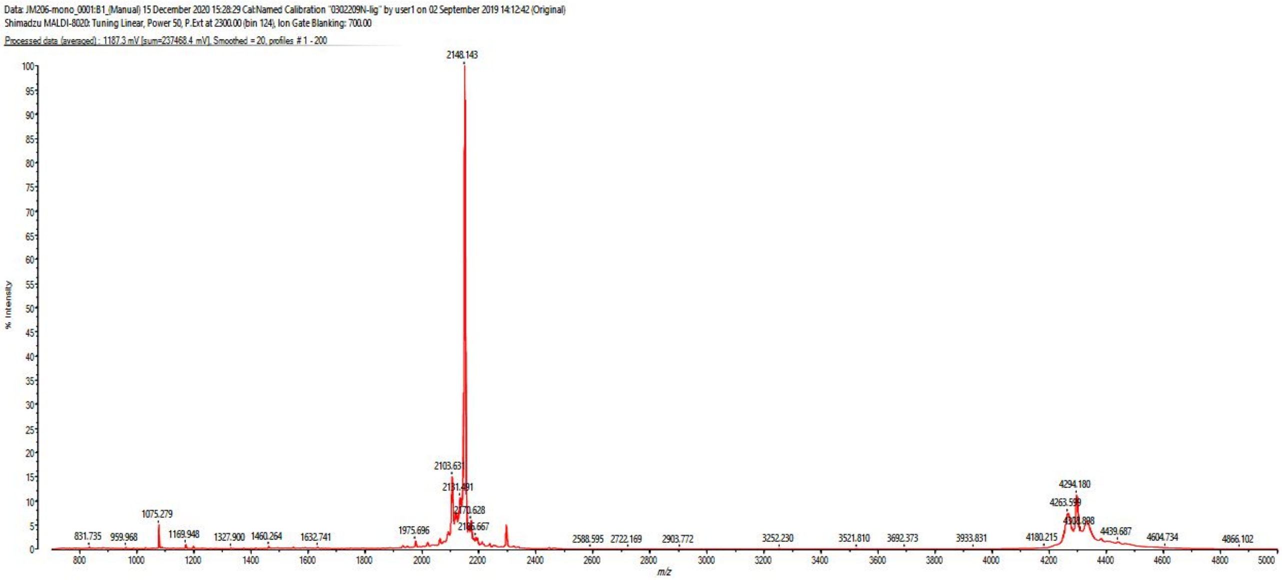

Figure S14. MALDI-TOF mass spectrum ^{nat}Lu-**1**-monomer (peak 1)

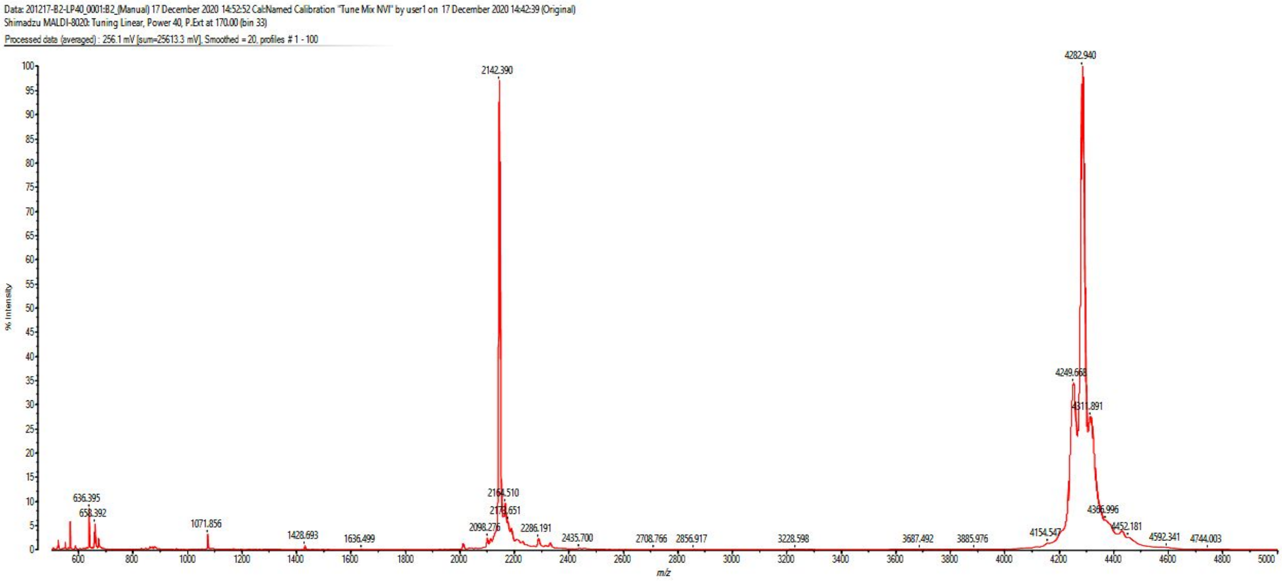

Figure S15. MALDI-TOF mass spectrum ^{nat}Lu-**1**-dimer (peak 2)

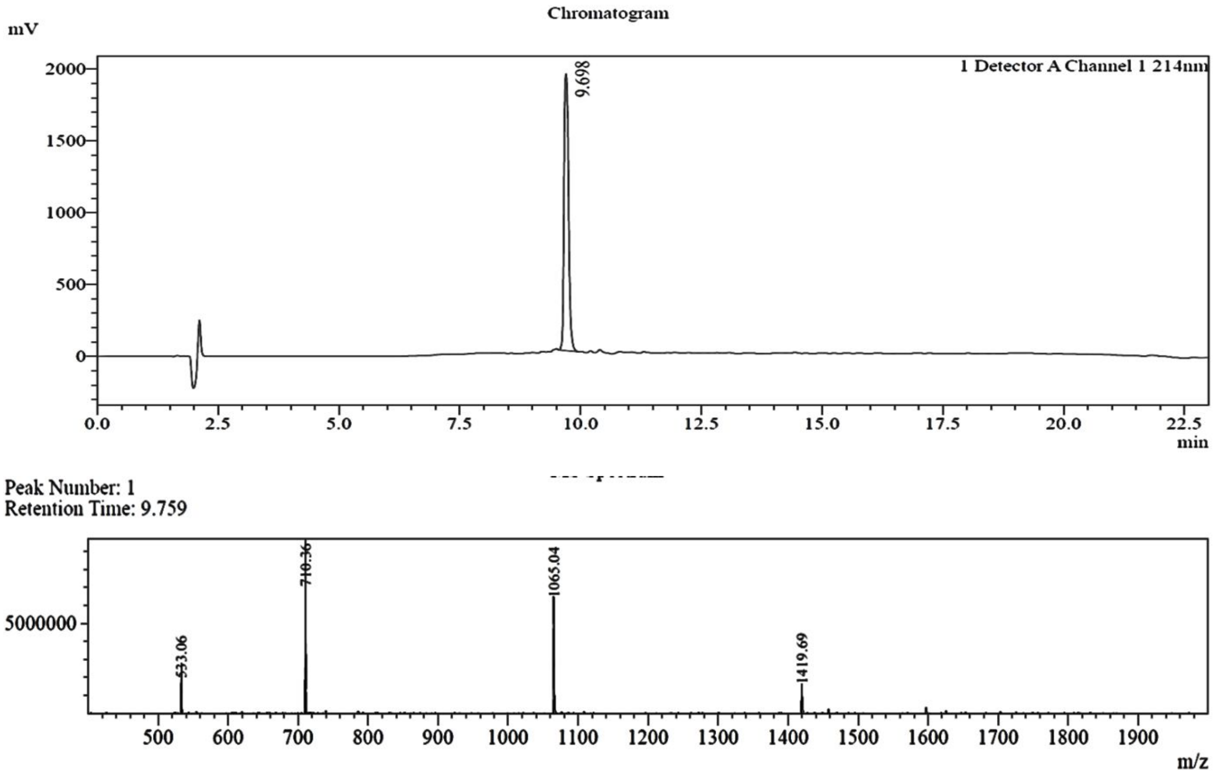

Figure S16. LC-MS profile of ^{nat}Lu-**2**

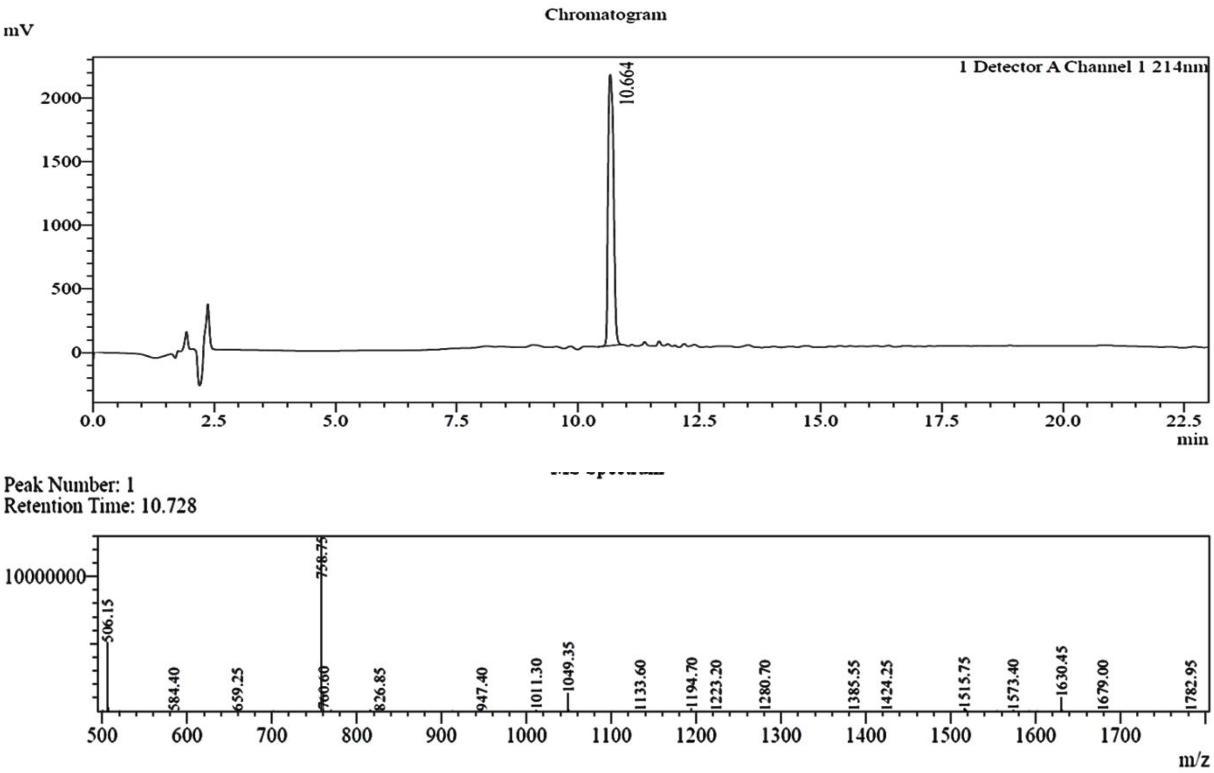

Figure S17. LC-MS profile of ^{nat}Lu-**5**

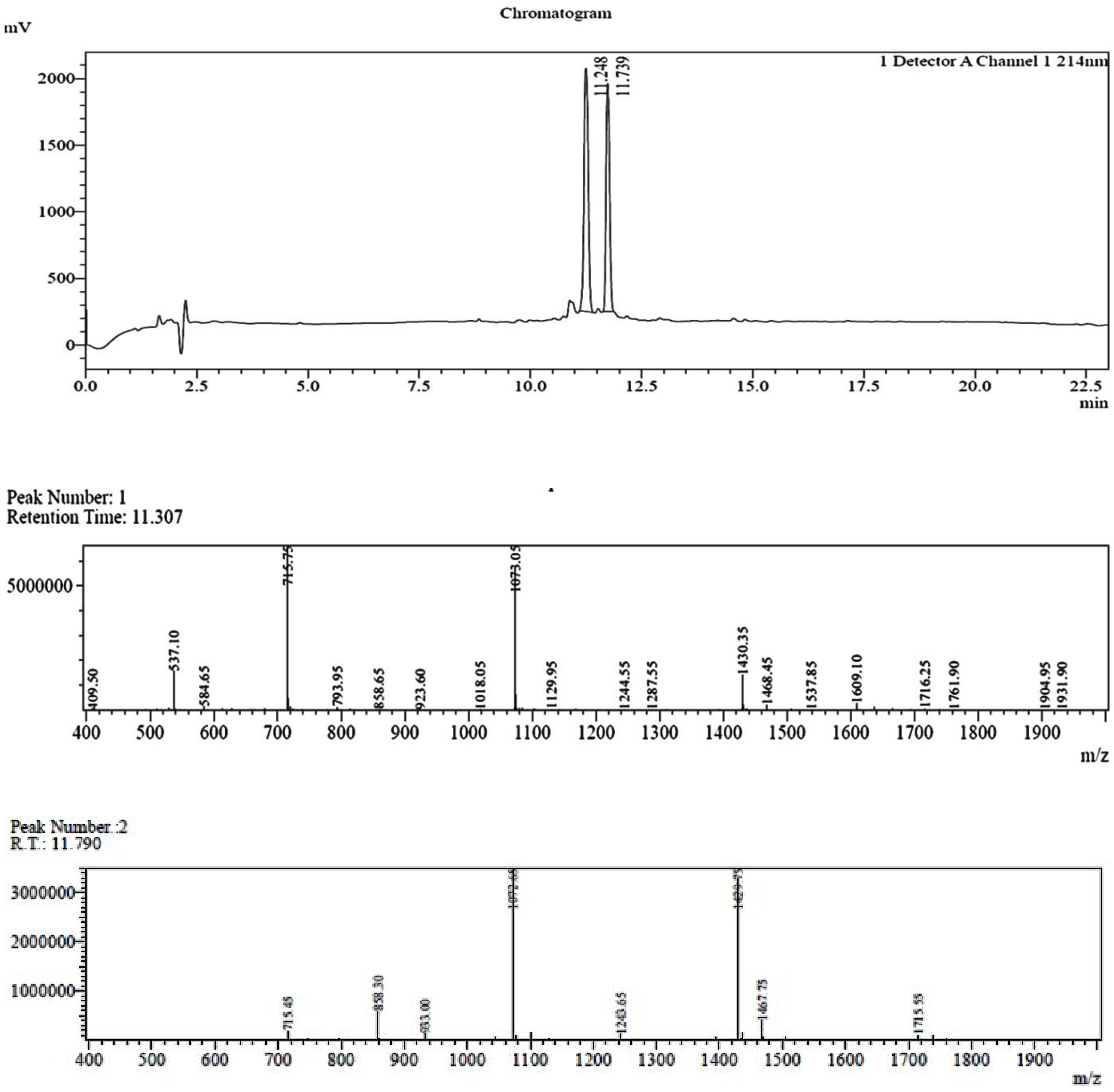

Figure S18. LC-MS profile of ^{nat}Lu-**6**

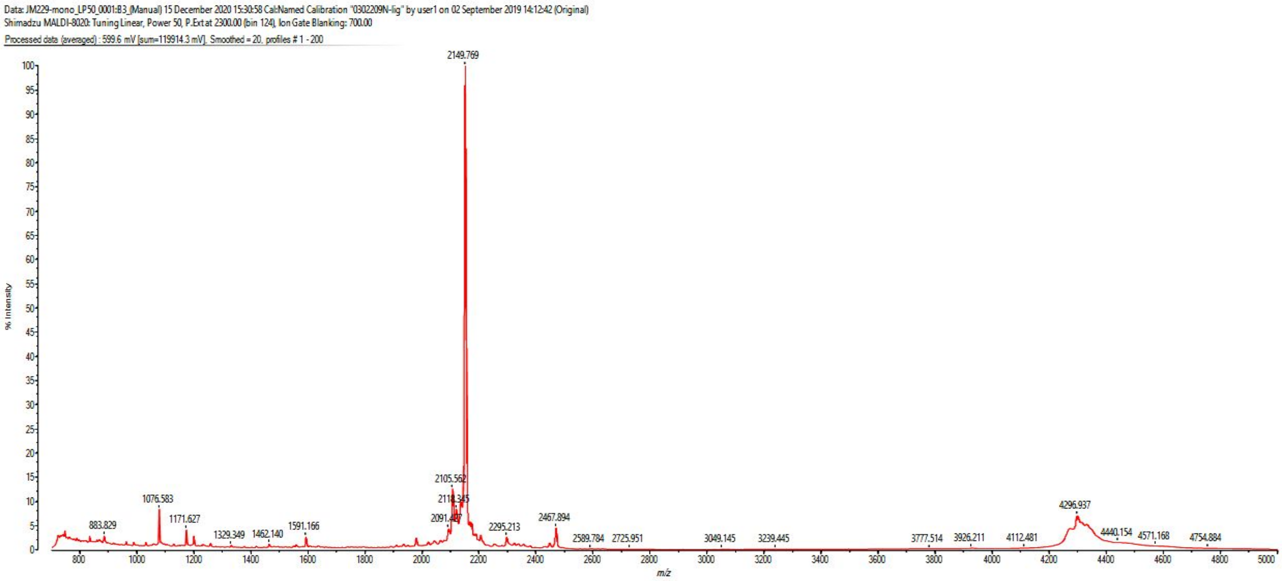

Figure S19. MALDI-TOF mass spectrum ^{nat}Lu-**6**-monomer (peak 1)

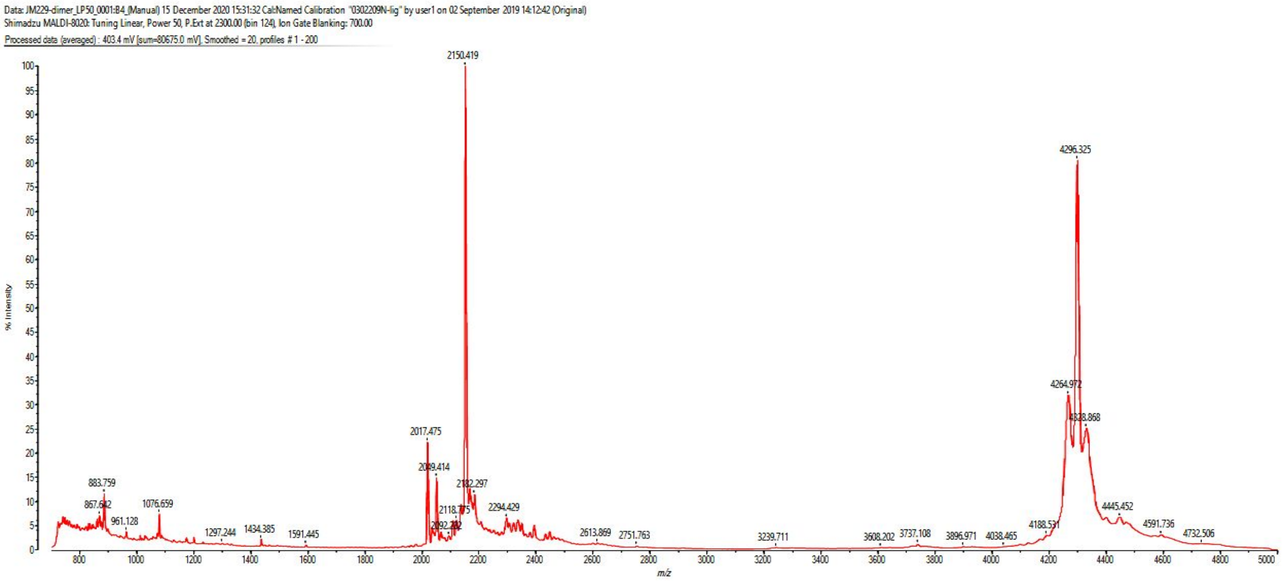

Figure S20. MALDI-TOF mass spectrum ^{nat}Lu-**6**-dimer (peak 2)

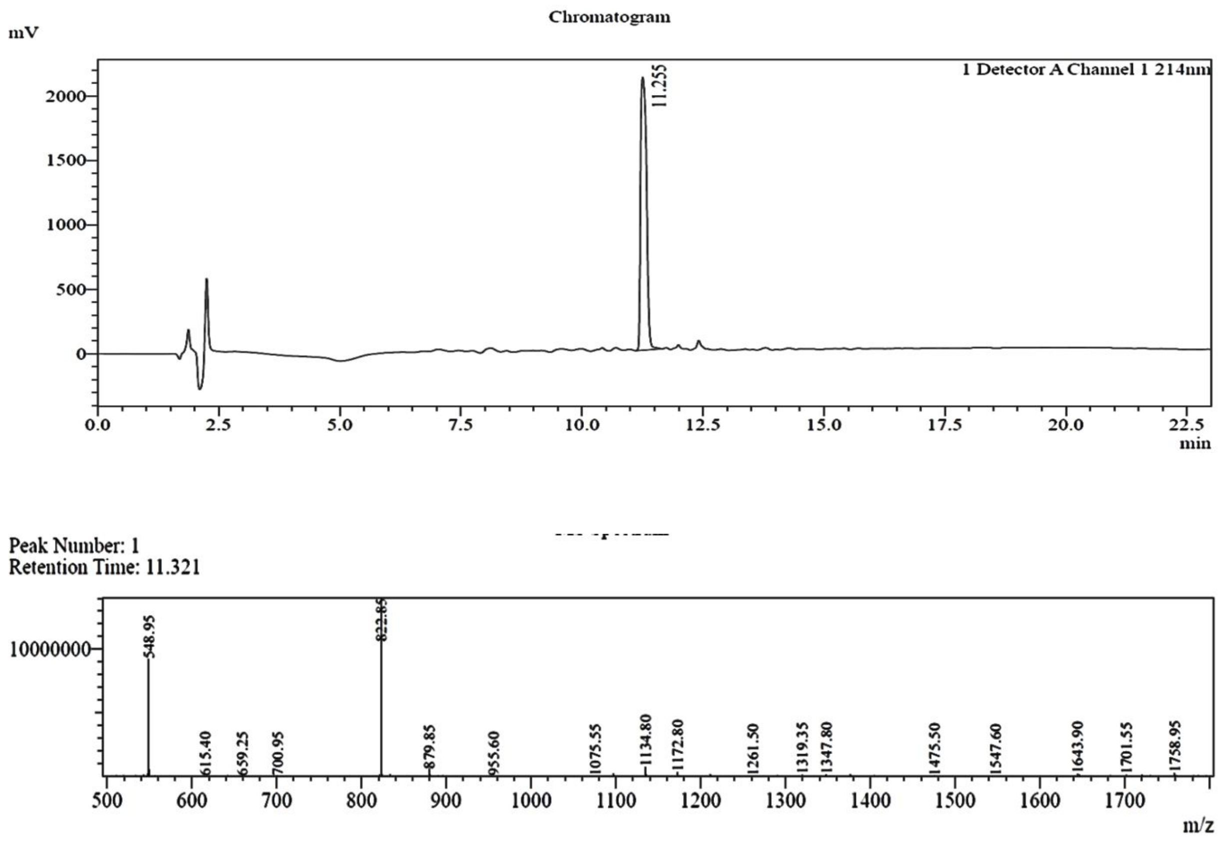

Figure S21. LC-MS profile of ^{nat}Lu-**7**

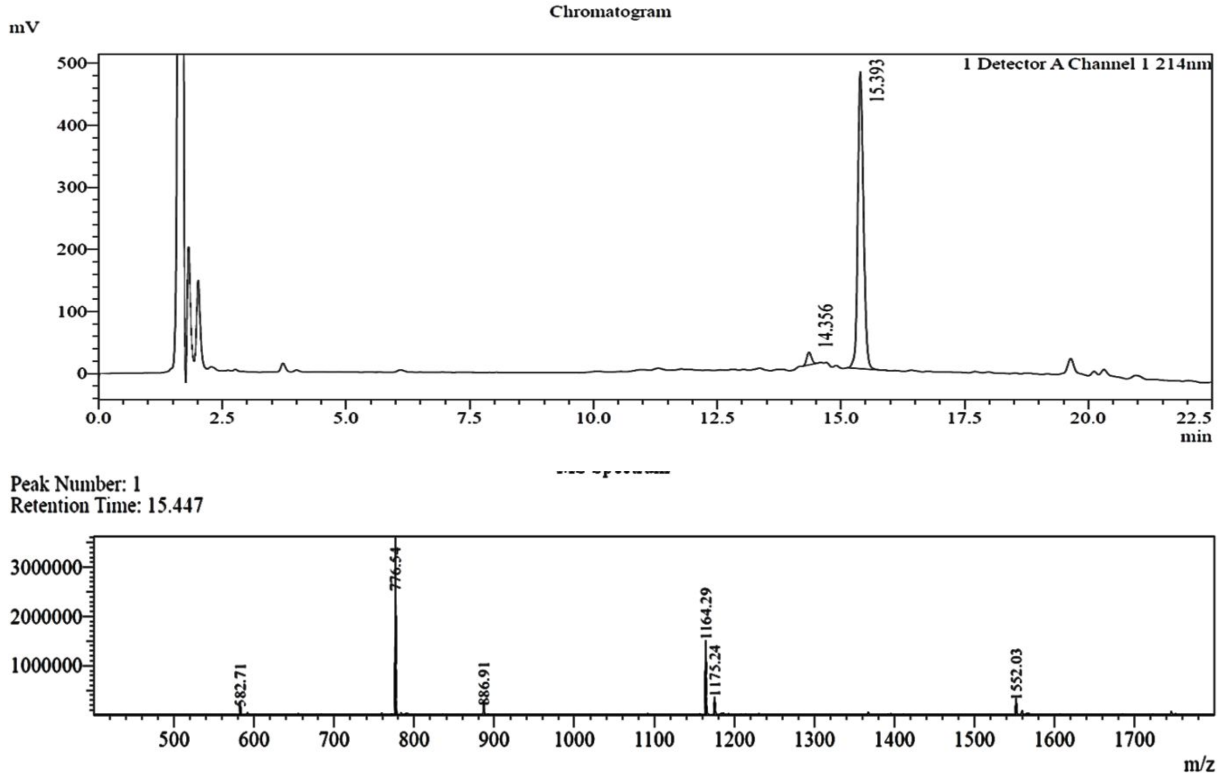

Figure S22. LC-MS profile of ^{nat}Lu-**8**

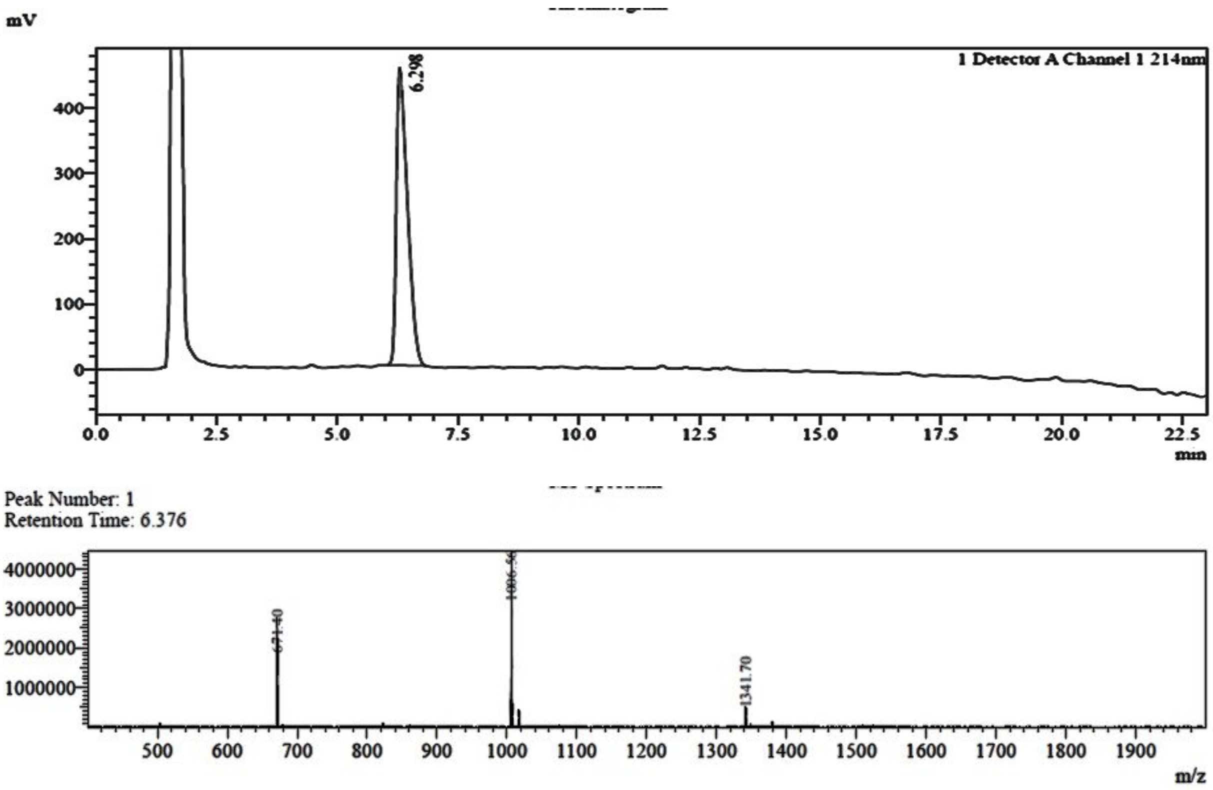

Figure S23. LC-MS profile of ^{nat}Lu-**9**

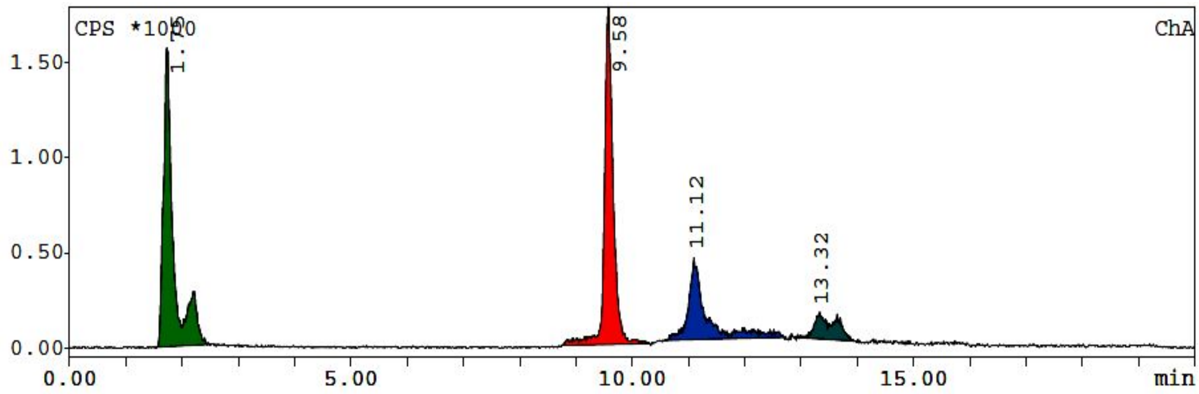

Figure S24. Radio-chromatogram of ^{177}Lu-**8**

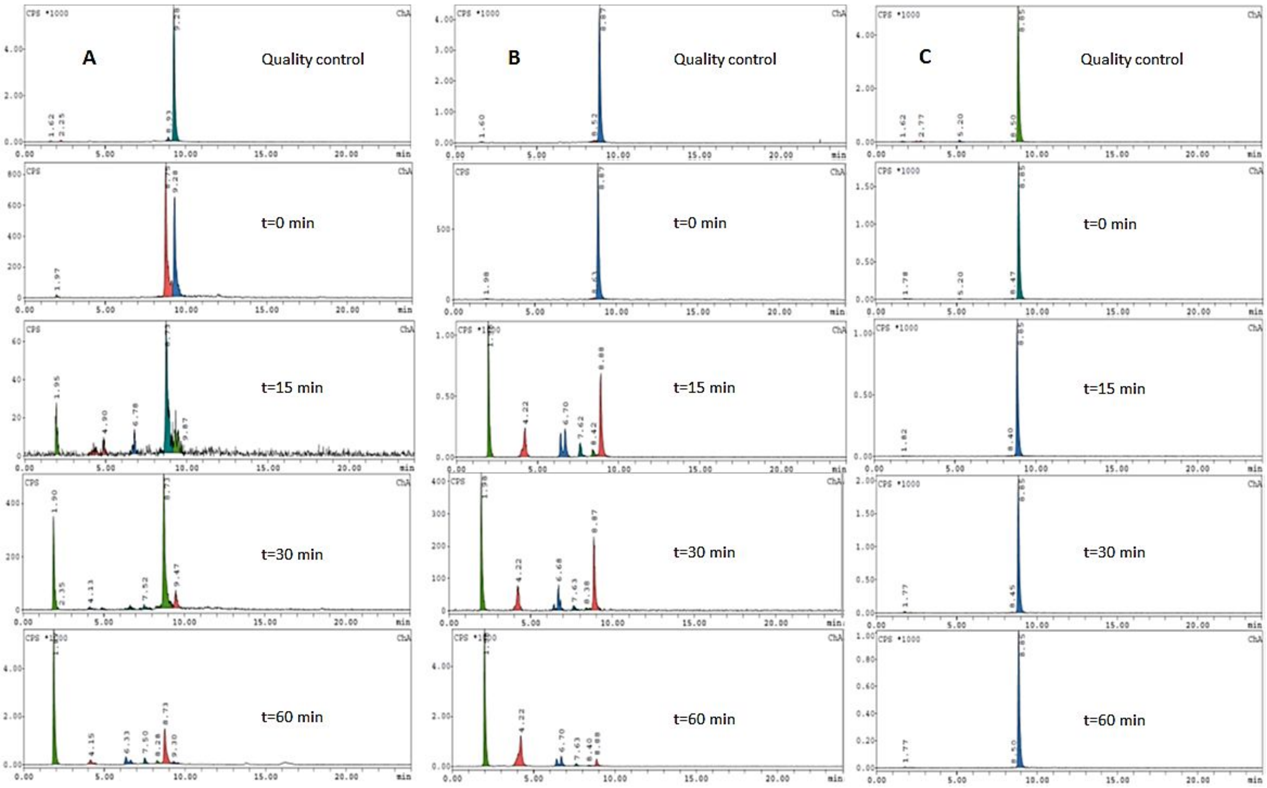

Figure S25. Representative chromatograms of *in vitro* metabolic stability study of A)^{177}Lu-**1** B)^{177}Lu-**2** and C) ^{177}Lu-**7** in human plasma over time.

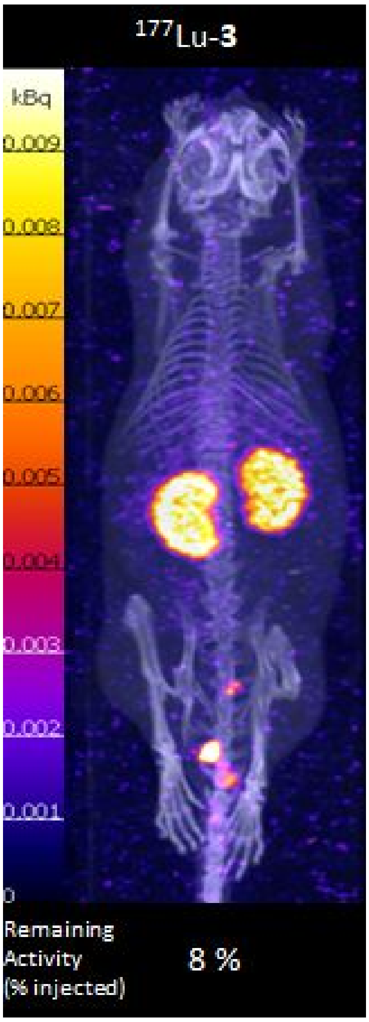

Figure S26. SPECT/CT images as maximum intensity projections (MIPs) of Jurkat tumor-bearing mice at 1h p.i. of ^{177}Lu-**3** (200 pmol, 15 MBq). The remaining activity in the body of the mice after 1 h (% of the remaining activity) is reported.
